# DHX15-independent roles for TFIP11 in U6 snRNA modification, U4/U6.U5 tri-snRNP assembly and pre-mRNA splicing fidelity

**DOI:** 10.1038/s41467-021-26932-2

**Published:** 2021-11-17

**Authors:** Amandine Duchemin, Tina O’Grady, Sarah Hanache, Agnès Mereau, Marc Thiry, Ludivine Wacheul, Catherine Michaux, Eric Perpète, Eric Hervouet, Paul Peixoto, Felix G. M. Ernst, Yann Audic, Franck Dequiedt, Denis L. J. Lafontaine, Denis Mottet

**Affiliations:** 1 GIGA-Molecular Biology of Diseases, Gene Expression and Cancer Laboratory, B34 +1, University of Liege, Avenue de l’Hôpital 1, B-4000 Liège, Belgium; 2 CNRS, Institut de Génétique et Développement de Rennes (IGDR) UMR 6290, Université de Rennes, F-35043 Rennes, France; 3 GIGA-Neurosciences, Unit of Cell Biology, B36 + 1, University of Liege, Avenue Hippocrate 15, B-4000 Liège, Belgium; 4 RNA Molecular Biology, Fonds de la Recherche Scientifique (F.R.S./FNRS), Université Libre de Bruxelles (ULB) - Biopark campus Gosselies, B-6041 Gosselies, Belgium; 5 NARILIS, ILEE and NISM Institutes, Laboratory of Physical Chemistry of Biomolecules, University of Namur, Rue de Bruxelles 61, B-5000 Namur, Belgium; 6 INSERM UMR 1098 RIGHT, EPIgenetics and GENe EXPression Technical Platform (EPIGENExp), INSERM, EFS-BFC, University Bourgogne Franche-Comté, F-25000 Besançon, France

**Keywords:** RNA, Non-coding RNAs, RNA metabolism

## Abstract

The U6 snRNA, the core catalytic component of the spliceosome, is extensively modified post-transcriptionally, with 2’-O-methylation being most common. However, how U6 2’-O-methylation is regulated remains largely unknown. Here we report that TFIP11, the human homolog of the yeast spliceosome disassembly factor Ntr1, localizes to nucleoli and Cajal Bodies and is essential for the 2’-O-methylation of U6. Mechanistically, we demonstrate that TFIP11 knockdown reduces the association of U6 snRNA with fibrillarin and associated snoRNAs, therefore altering U6 2′-O-methylation. We show U6 snRNA hypomethylation is associated with changes in assembly of the U4/U6.U5 tri-snRNP leading to defects in spliceosome assembly and alterations in splicing fidelity. Strikingly, this function of TFIP11 is independent of the RNA helicase DHX15, its known partner in yeast. In sum, our study demonstrates an unrecognized function for TFIP11 in U6 snRNP modification and U4/U6.U5 tri-snRNP assembly, identifying TFIP11 as a critical spliceosome assembly regulator.

## Abstract (web summary)

In yeast, TFIP11 and DHX15 promote the disassembly of spliceosome complex after splicing is completed. Here the authors show that human TFIP11 functions independently of DHX15 and is required for U6 snRNA 2’-O-methylation and U4/U6.U5 tri-snRNP assembly.

## Introduction

Splicing of precursor messenger RNA (pre-mRNA) is a fundamental process in eukaryotic gene expression. Unsurprisingly, alterations of pre-mRNA splicing have been implicated in multiple diseases, including neuromuscular, psychiatric, and X-linked disorders. In addition, dysregulation of splicing contributes significantly to hallmarks of cancer and thus represents a susceptibility that anti-cancer interventions can target^1^.

The splicing of introns in pre-mRNA is carried out by the spliceosome, a dynamic macromolecular complex composed of five small ribonucleoproteins (snRNPs). Each snRNP consists of a single small nuclear RNA (the U1, U2, U4, U5 and U6 snRNAs) associated with numerous proteins^2^. The biogenesis and maturation of snRNPs is a sequential multistep process that takes place in several subcellular compartments. After transcription in the nucleoplasm, U1, U2, U4 and U5 snRNAs are exported to the cytoplasm where the SMN complex promotes their association with Sm proteins to form the core U snRNP complexes^3^. Then, the SMN complex reimports the U snRNPs into nuclear Cajal Bodies (CBs) where they undergo site-specific post-transcriptional modifications directed by scaRNAs (small Cajal Body RNAs), a specialized class of snoRNAs (small nucleolar RNAs)^4^. Unlike the Sm-class U snRNAs, U6 snRNA assembly is confined to the nucleus. U6 undergoes site-specific post-transcriptional modifications by snoRNAs in the nucleolus rather than in CBs^5^. It is thought that modifications of U snRNAs may influence numerous aspects of pre-mRNA splicing including the interaction of U snRNAs with each other, interaction of U snRNAs with spliceosomal proteins, and U snRNP assembly, stability, and catalytic activity in the spliceosome. Except for U2 snRNA, there are few experimental studies demonstrating how these modifications contribute to U snRNA function and spliceosome activation in human cells^6^.

Two major classes of sca/snoRNAs guide site-specific post-transcriptional modifications of U snRNAs. Box C/D snoRNAs associate with a set of four core proteins (SNU13, NOP56, NOP58 and fibrillarin (FBL)) and carry out site-specific 2′-O-methylation of RNA. Box H/ACA snoRNAs associate with a different set of core proteins (NHP2, NOP10, GAR1 and DKC1) and control site-specific pseudouridylation of RNA^7^. In addition to the core proteins, sca/snoRNP assembly probably requires numerous assembly factors that associate transiently with the maturing particles to ensure the efficiency, specificity and quality control of sca/snoRNP production^8^. However, the full set of snoRNP assembly factors has not yet been fully identified.

A final step of regulation in snRNP biogenesis is the assembly of U4/U6 di-snRNPs and U4/U6.U5 tri-snRNPs in CBs. U4/U6 di-snRNPs are assembled in a process involving base-pairing between U4 and U6 and the association of proteins including SART3/p110 factor, Prp4K and Prp31^9^. The recruitment of U5 snRNP to the U4/U6 di-snRNP is mediated by protein-protein interactions, notably between Prp31 and the U5-specific Prp6^10^, ultimately leading to a stable ribonucleoprotein particle composed of 30 distinct proteins including SNRNP200, EFTUD2 and Prp8^9,11^. Nevertheless, the molecular events regulating this process remain unclear.

U snRNPs must be reused and reassembled in CBs at each round of splicing. This snRNP recycling is intrinsically dependent upon spliceosome disassembly. Our current knowledge of this essential step is mostly derived from work conducted in budding yeast. The Saccharomyces cerevisiae splicing factors Ntr1 (known as TFIP11 or STIP in human) and Ntr2 form the NTR complex that promotes the disassembly of U2, U5 and U6 through direct interaction with the RNA helicase Prp43 (known as DHX15 in human)^12–15^. However, no clear ortholog of yeast Ntr2 has been identified in humans, raising questions about the involvement of the TFIP11-DHX15 complex in human spliceosome regulation. In addition, whether TFIP11 and DHX15 might be implicated in the snRNP recycling phase after spliceosome disassembly is an open question.

In this study, we show that TFIP11 is critically important for 2’-O methylation of the U6 snRNA in the nucleolus. Upon depletion of TFIP11, the assembly of U4/U6.U5 tri-snRNPs is impaired and the fidelity of pre-mRNA splicing affected, leading to dramatic consequences for genome stability maintenance.

## Results

### TFIP11 localizes in nuclear speckles, Cajal Bodies and nucleoli

**Fig. 1:**
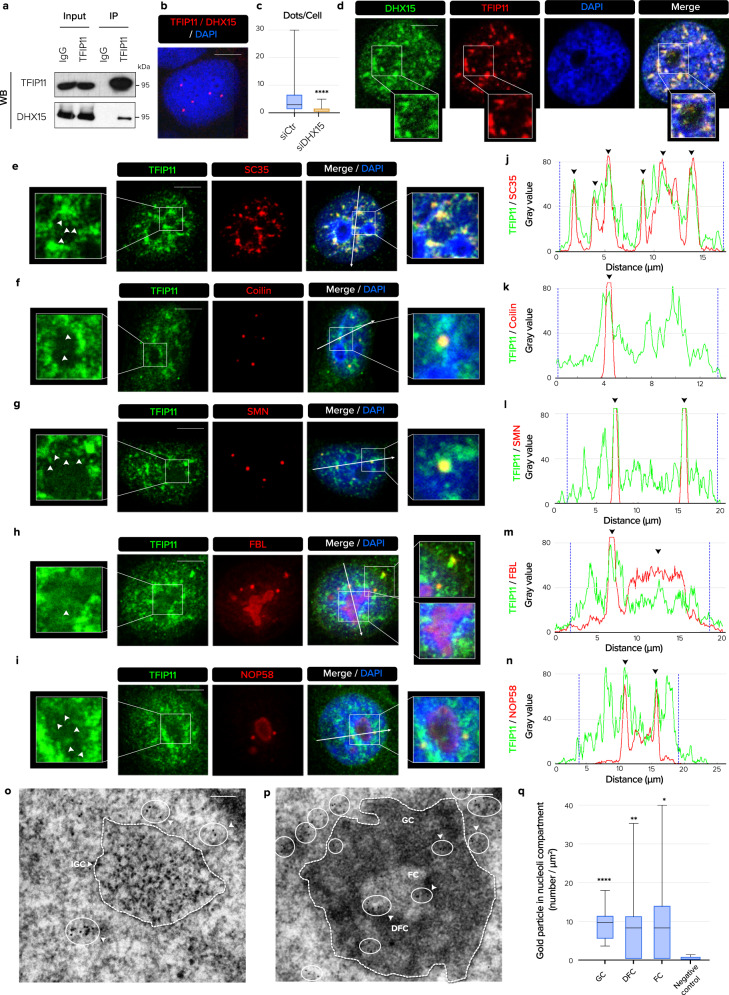
TFIP11 localizes in nuclear speckles, Cajal Bodies (CBs) and nucleoli. **a** Immunoprecipitation (IP) of endogenous TFIP11 from HeLa cells followed by western blotting (WB) for indicated proteins. **b** Representative image of endogenous TFIP11 and DHX15 interaction (in red) assessed by in situ proximity ligation assay (iPLA). Nucleus was counterstained in blue. Scale bar = 5 µm. **c** Quantification of TFIP11/DHX15 interaction detected by iPLA in HeLa cells transfected for 48 h with control siRNA (siCtr *n* = 293) or DHX15 siRNA (siDHX15 #1 *n* = 38). Box limits = 25th to 75th percentiles; line = median; whiskers = min to max. *p*-value calculated using unpaired two-tailed *t*-test (*p* < 0.0001). **d** HeLa cells co-stained with anti-DHX15 (in green) and anti-TFIP11 (in red) antibodies. Nucleus was counterstained in blue. Individual channels, merged channels and magnification of boxed regions are shown. Scale bar = 10 µm. **e**–**n** HeLa cells co-stained for TFIP11 (in green) and SC35 (**e**), coilin (**f**), SMN (**g**), FBL (**h**), or NOP58 (**i**) (in red). Individual channels, merged channels with nuclear staining (in blue) and magnification of boxed regions are shown. Scale bar = 5 µm. White arrows indicate diffuse nucleolar staining for TFIP11 with occasional more intense foci. **j**–**n** Intensity plots for regions in **e**–**i** (white arrows) indicate co-localization of TFIP11 with each corresponding protein (black arrows). Blue dashed lines indicate nucleus edges. **o**–**p** Endogenous TFIP11 in U2OS cells detected by immunogold/electron microscopy (EM). White arrows in (**o**) show 6-nm gold particles in white circles, indicating TFIP11 in perichromatin fibrils (PF) at the periphery of interchromatin granule clusters (IGC, white dashed line). Scale bar = 0.25 µm. White arrows in **p** indicate particles labeling TFIP11 in nucleolar compartments: dense fibrillar center (DFC), fibrillar center (FC) and granular component (GC). TFIP11 is also at the periphery of the nucleolus, delimited by the white dashed line. Scale bar = 0.25 µm. **q** Quantification of immunogold-labeled TFIP11 in the DFC, FC and GC in U2OS cells. Negative control = no primary antibody. *N* = 15 different nucleolar fields from 15 different cells. *p*-value calculated using two-tailed paired *t*-test. GC *p* < 0.0001; DFC *p* = 0.0035; FC *p* = 0.0107. Box limits = 25th to 75th percentiles; line = median; whiskers = min to max. Source data are provided as a Source Data file.

In yeast, several studies have shown that Ntr1 is a G-patch protein that binds and activates the ATP-dependent DEAH-Box RNA helicase Prp43 to catalyze the disassembly of the lariat-spliceosome^12–15^. However, the physical and functional interaction between TFIP11 and DHX15, the human protein counterparts of Ntr1 and Prp43, remains poorly characterized. First, we demonstrated that these two proteins interact in human cells by co-immunoprecipitation of the endogenous factors (Fig. 1a) and in situ proximity ligation assay (iPLA) experiments (Fig. 1b, c). Red fluorescent iPLA dots, indicating nanometer-range interaction between TFIP11 and DHX15 were detected in cell nuclei (Fig. 1b). No dots were detected when TFIP11 was depleted by siRNA, attesting to the specificity of this assay (Fig. 1c). Immunofluorescence (IF) further demonstrated that TFIP11 and DHX15 largely co-localized within the nucleus (Fig. 1d). Interestingly, higher magnification of the nucleolus showed the presence of a certain amount of TFIP11 in this compartment, in particular in the form of small foci. To further characterize the intranuclear localization of these two proteins, co-staining was performed with specific markers of different subnuclear bodies including nuclear speckles (NS), Cajal Bodies (CBs), Gemini of Cajal Bodies (GEMs) and nucleoli. TFIP11 co-localized with SC35, a marker of NS (Fig. 1e, f). TFIP11 was observed in coilin-positive CBs as well as in SMN-positive GEMs overlapping the CBs (Fig. 1f, l). Co-staining with FBL or NOP58 proteins in foci outside of the nucleolus confirmed the localization of TFIP11 in CBs (Fig. 1h, n). Again, higher magnification of the nucleolus confirmed the diffuse staining for TFIP11 with occasional more intense foci (white arrows) (Fig. 1e to n). DHX15 staining was observed in the nucleolus as previously shown^16^ and also in nuclear speckles. However, no DHX15 was observed in CBs or GEMs, providing initial insight that TFIP11 might have DHX15-independent nuclear functions (Supplementary Fig. 1).

To gain further insight into the localization of TFIP11, immunoelectron microscopy (EM) was performed. Endogenous TFIP11 was seen to be located on perichromatin fibrils (PF) at the periphery of the interchromatin granule cluster (IGC). These PF are considered to be the active sites of pre-mRNA transcription and processing such as splicing^17^. These observations suggest that TFIP11 was mostly present in active spliceosomes rather than as a “reserve” of splicing factors accumulated within the interchromatin granule cluster (Fig. 1o). However, no significant TFIP11 labeling was observed in the matrix of the CBs using immunoelectron microscopy. Interestingly, TFIP11 was found at the periphery of the nucleolus as well as in the three subcompartments of the nucleolus: the fibrillar center (FC), the dense fibrillar component (DFC), and the granular component (GC). A more precise quantification revealed that TFIP11 was statistically significantly more detected in the GC compartment than in the two other compartments (Fig. 1p, q). The nucleolar labeling was highly specific, as demonstrated by inspecting control sections on which the primary antibody was omitted (Supplementary Fig. 2). Together, these data suggest that TFIP11 might be important for the structure and/or function of nucleoli and CBs.

### TFIP11 interacts with coilin via its N-terminal IDP region

**Fig. 2:**
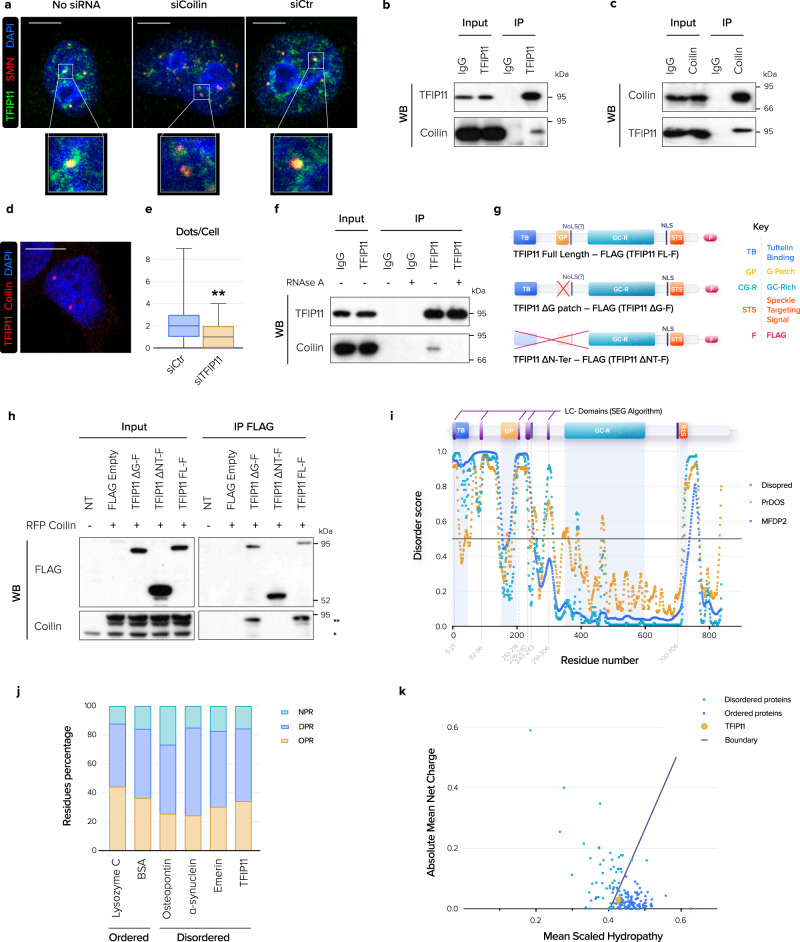
TFIP11 interacts with coilin via its disordered N-terminal region. **a** HeLa cells were mock-transfected (No siRNA) or transfected with coilin siRNA (siCoilin) or control siRNA (siCtr) for 48 h. Co-staining of SMN (in red) and TFIP11 (in green) was performed. Merged channels with nuclear staining (in blue) and magnification of boxed regions are shown. Scale bar = 5 µm. **b** Immunoprecipitation (IP) of endogenous TFIP11 from HeLa cells followed by western blotting (WB) for the indicated proteins. **c** Immunoprecipitation (IP) of endogenous coilin from HeLa cells followed by western blotting (WB) for the indicated proteins. **d** Representative image of the interaction (in red) of endogenous TFIP11 and coilin assessed by in situ proximity ligation assay (iPLA). Nucleus was counterstained in blue. Scale bar = 5 µm. **e** Quantification of TFIP11/coilin interaction detected by iPLA in HeLa cells transfected with control siRNA (siCtr *n* = 90) or TFIP11 siRNA (siTFIP11 #1 *n* = 70) for 48 h. *p*-value calculated using unpaired two-tailed *t*-test (*p* = 0.0020). Box limits = 25th to 75th percentiles; line = median; whiskers = min to max. **f** Immunoprecipitation (IP) of endogenous TFIP11 from HeLa cell extracts pre-treated or not with RNAse A followed by western blotting (WB) for the indicated proteins. **g** Schematic representation of the TFIP11-FLAG constructs used in co-immunoprecipitation experiments. **h** HeLa cells were co-transfected with RFP-coilin and the TFIP11-FLAG constructs schematized in **g** and immunoprecipitation (IP) was performed with anti-FLAG antibodies followed by western blotting (WB) for the indicated proteins. (*) endogenous coilin. (**) exogenous RFP-coilin. **i** Disorder prediction along the TFIP11 sequence by three algorithms: MFDp2 (Biomine), PrDOS and DISOPRED. The threshold score for disorder is 0.5 (solid black line). **j** Occurrence of OPR (order-promoting residues), DPR (disorder-promoting residues) and NPR (non-promoting disorder/order residues) in the sequence of well-ordered proteins (lysozyme C, BSA), known IDPs (Osteopontin, α-Synuclein, Emerin) and TFIP11. **k** Charge-Hydropathy plot using the PONDR algorithm. The solid black line represents the border between ordered and disordered phases. Source data are provided as a Source Data file.

Coilin is the main structural component of CBs, providing a scaffold for recruitment of other factors and CB assembly. As shown previously, coilin knockdown disrupted CBs, inducing formation of multiple small SMN-containing foci^18^. TFIP11 was poorly recruited to these residual CBs lacking coilin, suggesting that the presence of TFIP11 in CBs is dependent on coilin (Fig. 2a and Supplementary Fig. 3). Based on these observations, we asked whether TFIP11 interacted with coilin. We found that endogenous TFIP11 and coilin co-immunoprecipitate together (Fig. 2b, c). In addition, we detected TFIP11-coilin iPLA foci in the nucleus, indicating that these proteins are indeed in close proximity (< 40 nm) (Fig. 2d, e), and these foci were significantly lost upon TFIP11 depletion, attesting to specificity (Fig. 2e). RNase treatment prior to immunoprecipitation abolished the TFIP11-coilin interaction, indicating it relies on RNA (Fig. 2f). Finally, deletion mutant analysis revealed that the N-terminal region of TFIP11, but not the G-patch domain only, was required for the interaction with coilin (Fig. 2g, h).

Interestingly, the N-terminal region of TFIP11 is predicted to be an intrinsically disordered region (IDR). Three independent algorithms identified the region upstream of the G-patch domain (residues 1–150) and a domain spanning residues 175 to 250 as IDRs. A C-terminal region (residues 720–770) was also predicted to be disordered (Fig. 2i). The TFIP11 sequence is depleted in so-called order-promoting residues (OPR, 34.2%) and enriched in disorder-promoting residues (DPR, 50.2%) (Fig. 2j), a feature shared among many well-known intrinsically disordered proteins (IDP). Typical features of IDPs also include low overall hydrophobicity and a large net charge, important for electrostatic interactions^19^. With a net charge of 0.0299 and a hydropathy of 0.4289, TFIP11 lies at the theoretical boundary between ordered and disordered proteins in the Charge-Hydropathy plot, close to similar IDPs bearing a low mean net charge at neutral pH and a hydropathy close to average (Fig. 2j). In addition, TFIP11 has an Rg value of 96 Å and an Rh value of 71 Å, giving an Rg/Rh ratio of 1.35. Such a ratio is typical of random coil and indicates that the protein probably lacks a fixed or ordered secondary/tertiary structure^20^. Finally, the SEG algorithm^21^ identifies five low complexity (LC) domains (residues 5–21; residues 82–96; residues 210–216; residues 226–240 and residues 291–306) overlapping the N-terminal IDR of TFIP11. Together these data suggest that TFIP11 is an IDP with LC domains, which might adopt multiple intermolecular conformations and interact weakly with numerous protein and/or RNA partners.

### TFIP11 knockdown alters Cajal Bodies, nuclear speckles and nucleolar structure

**Fig. 3:**
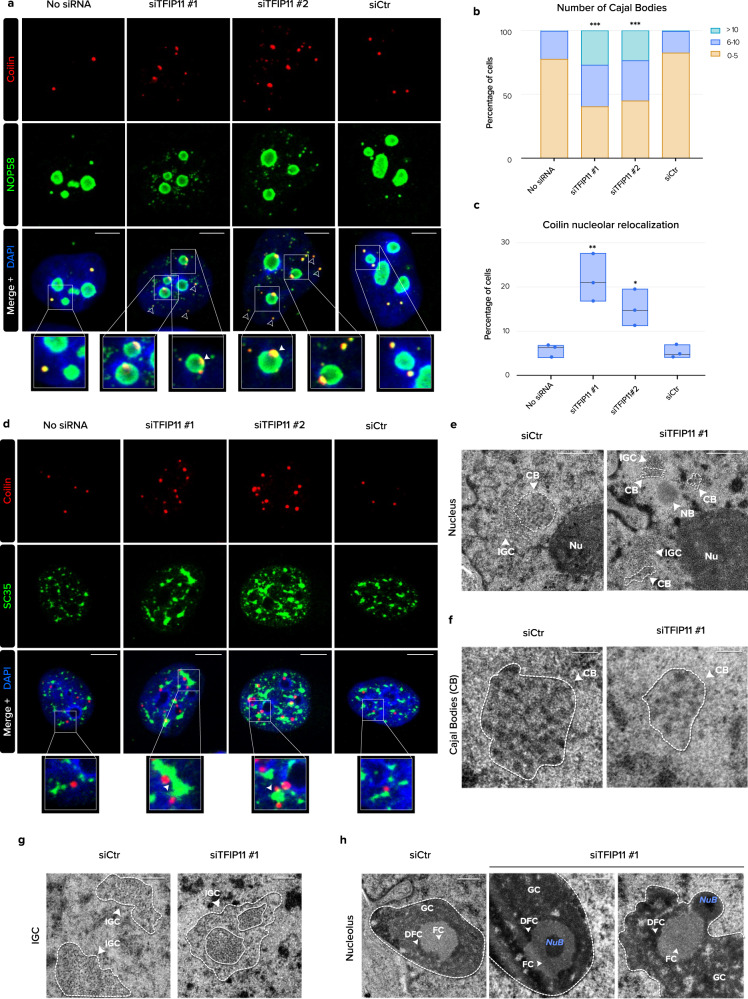
TFIP11 knockdown alters morphology of Cajal Bodies (CBs), nuclear speckles and nucleoli. **a** HeLa cells were mock-transfected (No siRNA) or transfected for 48 h with one of two different TFIP11 siRNAs (siTFIP11 #1 and siTFIP11 #2) or control siRNA (siCtr). Cells were co-stained with anti-coilin (in red) and anti-NOP58 (in green) antibodies. Individual channels and merged channel with nuclear staining (in blue) and magnification of boxed regions are shown. Scale bar = 5 µm. **b** Quantification of the number of Cajal Bodies (CBs) in HeLa cells transfected as in **a** (No siRNA *n* = 363/siTFIP11 #1 *n* = 247/siTFIP11 #2 *n* = 297/siCtr *n* = 289 from four independent experiments). **c** Quantification of the number of cells with coilin foci in the nucleolus (No siRNA *n* = 343/siTFIP11 #1 *n* = 540/siTFIP11 #2 *n* = 634/siCtr *n* = 431 from three independent experiments). *p*-values for panels **b**–**c** were calculated by Kruskal–Wallis test followed by Dunn post-hoc test with Benjamini–Hochberg correction. Adjusted *p*-values < 0.05 (relative to siCtr) are displayed. Box limits = min to max; line = median. **d** HeLa cells transfected as in **a** and co-stained with anti-coilin (in red) and anti-SC35 (in green) antibodies. Individual channels and merged channel with nuclear staining (in blue) and magnification of boxed regions are shown. Scale bar = 5 µm. **e**, **f** Transmission electron microscopy (TEM) showing Cajal Bodies (CB) of HeLa cells transfected for 48 h with control siRNA (siCtr) or TFIP11 siRNA (siTFIP11 #1). IGC = interchromatin granule clusters. Nu = nucleolus. NB = nuclear body. Scale bar = 0.25 µm. **g** TEM showing interchromatin granule clusters (IGC) of HeLa cells transfected as in **e**. Scale bar = 0.2 µm. **h** TEM showing nucleoli of HeLa cells transfected as in **e**. The three classical compartments (the dense fibrillar center–DFC, the fibrillar center–FC, and the granular component–GC) of the nucleolus (delimited by white dashed line) are indicated. NuB = Nucleolar Body. Scale bar = 0.2 µm. Source data are provided as a Source Data file.

IDPs can participate in the dynamic self-assembly of membrane-less nuclear compartments such as CBs, NS and nucleoli by liquid–liquid phase separation (LLPS)^22^. We first examined the impact of TFIP11 depletion on the morphology of CBs. In control cells, both coilin and SMN co-localized with NOP58, defining spherical CBs of roughly similar size and numbering between 1–5 in most cells. In TFIP11-depleted cells, we observed an increase in the number of coilin- and SMN-positive foci, which were also more heterogeneous in size (Fig. 3a, b). Remarkably, TFIP11 knockdown induced relocalization of coilin and SMN to the NOP58-positive dense fibrillar component of the nucleolus forming a cap-like structure (Fig. 3a–c and Supplementary Figs. 4–5), a phenotype frequently observed upon the knockdown of proteins involved in the assembly of snRNP complexes in CBs^23,24^. The striking coalescence of coilin and SMN with nucleoli observed upon TFIP11 depletion reflects the structural relationships between CBs and nucleoli^25^, and suggests the IDP domain of TFIP11 may contribute to condensate homeostasis in the nucleus. In agreement with this notion, TFIP11 depletion also resulted in enlarged and irregularly shaped SC35-positive NS located in the vicinity of CBs (Fig. 3d).

EM analysis revealed that TFIP11-depleted cells contained structures that exhibited the morphological characteristics of CBs, but were more heterogeneous in size and shape than control CBs (Fig. 3e, f). In TFIP11-depleted cells, examination of the ultrastructure of NS revealed that some parts of the IGC are more compacted, as evidenced by increased density of the interchromatinic substance (Fig. 3g, white line), and tend to fuse and group in clusters (Fig. 3g, white dashed line), an observation which is consistent with the increased SC35 intensity observed by IF (Fig. 3d). Finally, EM observation of nucleoli showed an additional electron-dense round structure, which was either contiguous with the dense fibrillar component, or within the fibrillar center, or at the periphery of the nucleolus (Fig. 3h, NuB). Altogether, these observations led us to hypothesize that TFIP11 may be important in regulating snRNP and/or sca/snoRNP assembly in biomolecular condensates such CBs, NS and nucleoli.

### TFIP11 binds U snRNAs, scaRNAs and snoRNAs

**Fig. 4:**
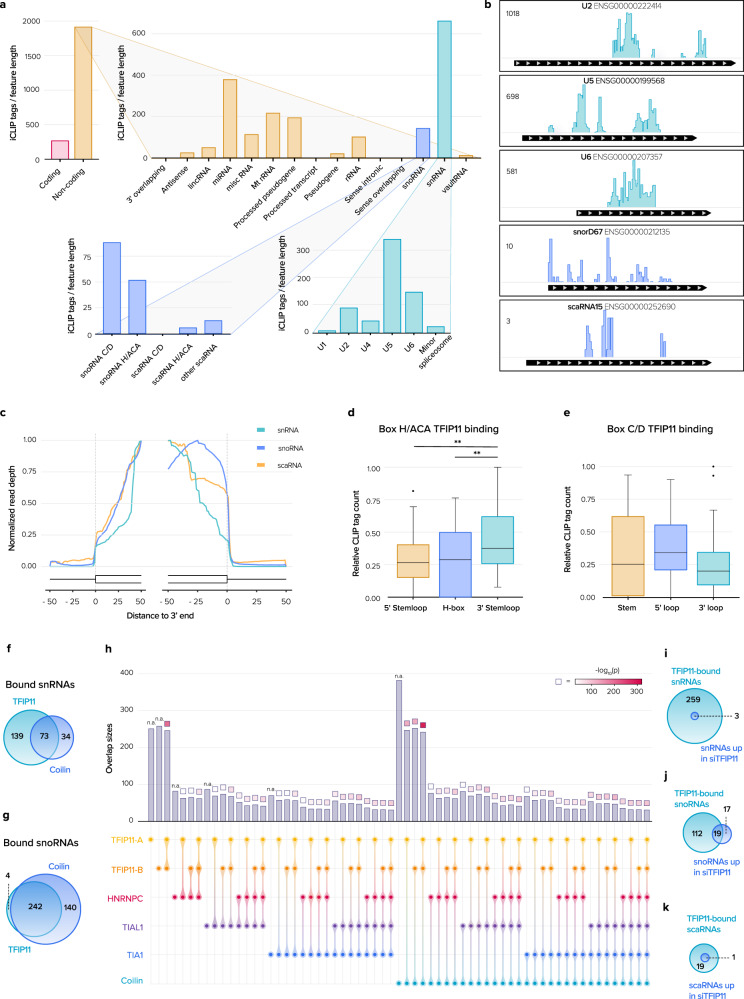
TFIP11 iCLIP identifies small non-coding RNAs as main targets. **a** Tags mapping to genes with significant iCLIP peaks in two independent libraries classified by transcript biotype. Bars represent number of iCLIP tags normalized by transcript length, averaged across libraries. Where transcripts can arise from multiple genomic locations (e.g., U6), all genomic locations are included. **b** Histograms represent TFIP11 iCLIP tag counts at significant binding sites (1 nt resolution) averaged across libraries for selected transcripts. Numbers on upper left of panels = tag count scale. **c** For transcripts of the noted type with mapped iCLIP tags, coverage at each nucleotide was summed, averaged across libraries and divided by the highest average number of tags mapping to displayed transcript region. Green = snRNA; blue = snoRNA; orange = scaRNA. **d**, **e** Distribution of TFIP11 iCLIP tags from two libraries across box H/ACA snoRNAs (overall *p* = 0.002 by one-way ANOVA; *p* = 0.006 for 3′ stemloop vs. 5′ stemloop and *p* = 0.005 for 3′ stemloop vs. H-box by Tukey post-hoc test, *n* = 10,498 tags mapping to 45 genes) (**d**) and box C/D snoRNAs (overall *p* = 0.15 by one-way ANOVA, *n* = 15,973 tags mapping to 51 genes) (**e**). Bands indicate medians, boxes indicate first and third quartiles and whiskers indicate 1.5× the interquartile range. **f**, **g** Venn diagrams of snRNAs (**f**) and snoRNAs (**g**) bound by TFIP11 and coilin. Where transcripts can arise from multiple genomic copies, each copy is treated separately. **h** UpsetR plot of snoRNAs bound to splicing factors (TIA1, TIAL1, HNRNPC), to coilin and to TFIP11 (two independent iCLIP libraries: TFIP11-A and TFIP11-B), to visualize the number of commonly bound snoRNAs. Columns represent the number of snoRNAs in the categories schematized beneath. Colored squares above each column represent enrichment *p*-value computed by SuperExactTest. Categories are not independent: e.g., the snoRNAs in the TFIP11-A category are also present in all other categories that include TFIP11-A. **i**–**k** Venn diagrams of snRNA (**i**), snoRNA (**j**) and scaRNA (**k**) targets of TFIP11 that increase upon TFIP11 knockdown 72 h post-transfection. Where transcripts can arise from multiple genomic copies, each copy is treated separately. Source data are provided as a Source Data file.

The presence of TFIP11 in CBs, NS and nucleoli suggested it might interact with a wide range of RNA substrates, potentially including U snRNAs, scaRNAs and snoRNAs. This was tested systematically by performing iCLIP^26^ (Supplementary Fig. 6). Interestingly, the highest enrichment of TFIP11 iCLIP-peaks was detected in non-coding RNAs, where iCLIP-peaks were found in U snRNAs and intron-encoded sca/snoRNAs, with peaks in both box H/ACA and box C/D sca/snoRNAs (Fig. 4a, b). iCLIP tags mapped almost exclusively inside the sca/snoRNA 5’ and 3’ end boundaries, suggesting that TFIP11 binds sca/snoRNAs after processing from introns (Fig. 4c). The distribution of iCLIP tags within the two families of sca/snoRNAs showed that TFIP11 preferentially binds to the 3’ stem loop structure of box H/ACA sca/snoRNAs (Fig. 4d). No preferred binding regions for TFIP11 were observed in box C/D sca/snoRNAs (Fig. 4e). Interestingly, our iCLIP data revealed more TFIP11 binding near the 5’ end of snoRNAs involved in U6 snRNA methylation than there is near the 5’ end of sno/scaRNAs that methylate other U snRNAs or pre-rRNAs, suggesting a distinct pattern of interaction and potential specificity towards snoRNAs that guide U6 snRNA modification (Supplementary Fig. 7). As TFIP11 and coilin co-localize and interact, we compared our TFIP11 iCLIP data with a published coilin iCLIP dataset^27^. Strikingly, TFIP11 and coilin bind a large number of common targets. Indeed, 63–68% targets associated with coilin were also bound by TFIP11 (Fig. 4f, g), suggesting a tight functional interaction between these two proteins. Such an overlapping binding profile was not observed for other nuclear RNA binding proteins such as TIA, TIAL1^28^ and HNRNPC^29^ (Fig. 4h). As knockdown of proteins that are essential for CB integrity can result in lower expression of all U snRNAs^30^, we assessed whether TFIP11 modulated U snRNA or sca/snoRNA levels by performing a small non-coding RNA-seq (sncRNA-seq) analysis. No ncRNAs showed significant decrease in expression upon TFIP11 knockdown. Inversely, only 40 out of 431 detected ncRNAs were increased (3 U snRNAs, 1 scaRNA and 36 snoRNAs). Of these 40 ncRNAs, only 23 are binding targets of TFIP11 (Fig. 4i to k and Supplementary Data 1). These data suggest that overall, the depletion of TFIP11 has very little effect on sn/snoRNA expression or metabolic stability.

### TFIP11 knockdown alters U4/U6.U5 tri-snRNP stability and spliceosome assembly

**Fig. 5:**
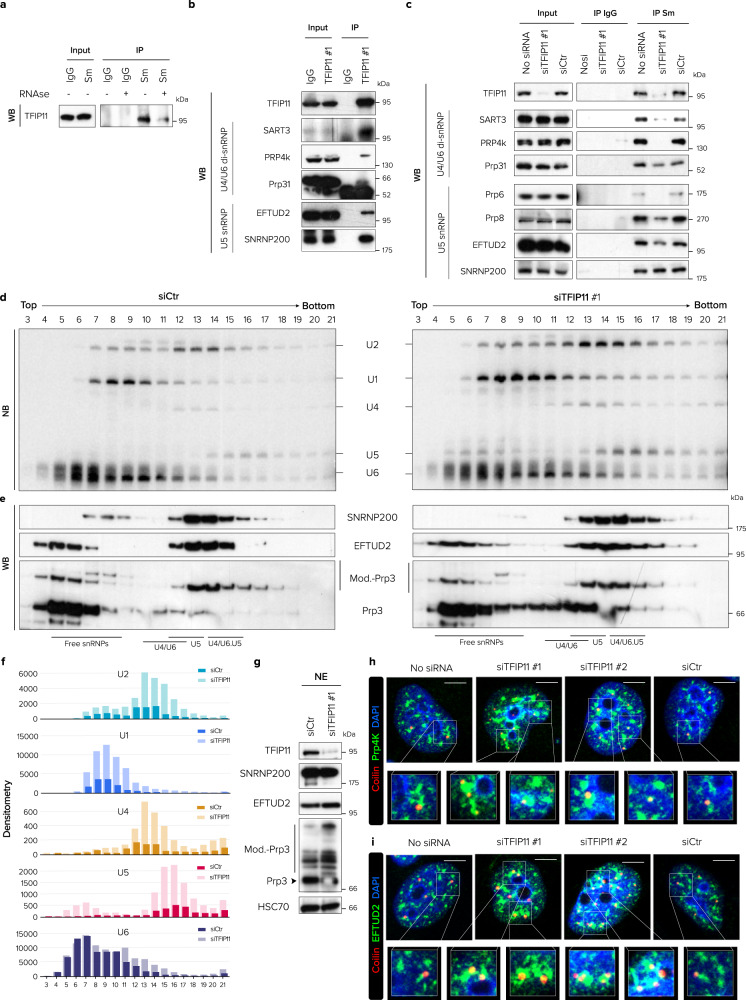
TFIP11 is required for U4/U6.U5 tri-snRNP stability. **a** Immunoprecipitation (IP) of endogenous Sm proteins from HeLa cell extracts pre-treated or not with RNAse A followed by western blotting (WB) for the indicated proteins. **b** Immunoprecipitation (IP) of endogenous TFIP11 from HeLa cell extracts followed by western blotting (WB) for the indicated snRNP-specific proteins. **c** Immunoprecipitation (IP) of endogenous Sm protein from extracts of HeLa cells mock-transfected (No siRNA) or transfected for 48 h with one TFIP11 siRNA (siTFIP11 #1) or control siRNA (siCtr) followed by western blotting (WB) of the indicated snRNP-specific proteins. **d**, **e** Glycerol-gradient fractionation. **d** Northern blotting (NB) analysis of U1, U2, U4, U5 and U6 snRNAs isolated from a glycerol-gradient (10–30%) fractionation of nuclear extracts from siCtr- and siTFIP11 #1-transfected cells 72 h post-transfection. NB data shown are representative of two independent experiments. **e** Western blotting (WB) analysis of indicated proteins isolated from the same glycerol-gradient (10–30%) fractions. **f** Densitometry scanning of NB autoradiographs for each U snRNA in each fraction of nuclear extracts from siCtr- and siTFIP11 #1-transfected cells. **g** Western blotting for the indicated proteins on the nuclear extracts (NE) of HeLa cells transfected for 72 h with control siRNA (siCtr) or with TFIP11 siRNA (siTFIP11 #1). **h**, **i** HeLa cells were mock-transfected (No siRNA) or transfected for 48 h with one of two different TFIP11 siRNAs (siTFIP11 #1 and siTFIP11 #2) or control siRNA (siCtr), then co-stained with anti-coilin antibody (in red) and antibodies against Prp4K (**h**) or EFTUD2 (**i**) (in green). Merged channels with nuclear staining (in blue) and magnification of boxed regions are shown. Scale bar = 5 µm. Source data are provided as a Source Data file.

Amongst U snRNAs, our iCLIP data showed that TFIP11 binds to U2, U4, U5 and U6 snRNAs (Fig. 4a). To determine whether TFIP11 was associated with U snRNAs bound by Sm proteins, we immunoprecipitated snRNPs using anti-Sm antibodies and analyzed proteins by western blotting. TFIP11 co-immunoprecipitated with Sm proteins but was not detected in the precipitate when RNA was degraded by RNase treatment, indicating that TFIP11 is associated with Sm-bound RNAs (Fig. 5a).

As TFIP11 localizes in CBs, the site where the U4/U6.U5 tri-snRNP is formed, we hypothesized that TFIP11 could be stably associated with components of the U4/U6 di-snRNP and of the U5 snRNP within the U4/U6.U5 tri-snRNP complex. To address this question, co-immunoprecipitation experiments were performed in a high-salt concentration buffer (500 mM NaCl). Under these stringent conditions, the tri-snRNP disassembles into separate U5 snRNP and U4/U6 di-snRNP particles^31,32^. In these conditions TFIP11 stably interacted with most of the U4/U6 di-snRNP-specific proteins, including SART3, Prp4K and Prp31, and also associated with the U5 snRNP-specific proteins EFTUD2 and SNRNP200 (Fig. 5b).

To assess if TFIP11 influences the strength of the interactions between snRNP‐specific proteins and U snRNA, RNP complexes were immunopurified in the stringent salt conditions described above using anti-Sm antibodies in the presence and absence of TFIP11. TFIP11 knockdown did not affect the abundance of SART3, Prp4K or EFTUD2 (Supplementary Fig. 8), but did substantially decrease the association of Sm-associated U4/U6 snRNA particles with SART3, Prp4K, and to a lesser extent Prp31. Interestingly, TFIP11 knockdown also impacted the salt-stable interaction^33^ between Sm-associated U5 snRNA and U5 snRNP-specific proteins Prp6, Prp8 and EFTUD2 while the association with SNRNP200 was not detectably affected. We conclude that TFIP11 plays a key role in regulating the assembly/stability of both U4/U6 di-snRNP and U5 snRNP particles (Fig. 5c).

To examine whether TFIP11 depletion also altered U4/U6.U5 tri-snRNP formation under physiological conditions, nuclear extracts from TFIP11-depleted and control cells were separated by glycerol-gradient ultracentrifugation under low-salt conditions (150 mM NaCl). Northern blotting analysis revealed that TFIP11 depletion led to a notable accumulation of U4/U6.U5 tri-snRNP (fractions 13–15) compared to control conditions. These tri-snRNPs also sedimented as a broader peak in fractions 16 to 19, suggesting that they may be more prone to aggregation. An increase in the amount of free U5 was observed in the upper fractions of the gradient (fractions 5 to 9). These observations suggest specific defects in the incorporation of individual U5 snRNP components into a maturing tri-snRNP particle and/or instability of this particle. Moreover, the accumulation of both U1 and U2 snRNAs in addition to U4, U5 and U6 snRNAs in fractions 17–19 suggests that the spliceosome can still assemble but is stalled in a B-like conformation (Fig. 5d–f). In parallel, analysis of protein distribution across the gradient showed that SNRNP200, the U5 snRNP-specific helicase required for U4/U6 unwinding and spliceosomal B to B^act^ transition, accumulated in fractions 15 to 17 (which contain tri-snRNPs) but was absent or under-represented in fractions 7–9. This suggests that SNRNP200 is more recruited to, or more stably associated, with B-like spliceosome intermediates and might not be efficiently recycled. EFTUD2, the co-factor that regulates the activity of SNRNP200, accumulates in the same fractions as SNRNP200 (fractions 15 to 17), confirming the formation of a stalled B-like spliceosome complex (Fig. 5e). Prp3, a protein required for U4/U6 di-snRNP and U4/U6.U5 tri-snRNP formation^34^, accumulated in fractions 11 and 12 (which contain U4/U6 di-snRNPs), but also in lower and higher fractions upon TFIP11 depletion. Ubiquitination and sumoylation of Prp3 are required for stabilization of the U4/U6.U5 tri-snRNP^35,36^, while de-ubiquitination/de-sumoylation cycles of Prp3 are required for the ejection of U4 snRNA during B to B^act^ transition. Post-translationally modified forms of Prp3 accumulate in TFIP11-depleted cells (Fig. 5g), which may further reflect alterations in the transition from B to B^act^ conformation of the spliceosome upon TFIP11 depletion^36^. Altogether, these data suggest that TFIP11 knockdown might cause structural rearrangement in the U4/U6.U5 tri-snRNP and alter the formation and/or stability of spliceosomal intermediates, impacting the dynamics and assembly of the spliceosome machinery.

As incomplete or immature tri-snRNP intermediates can accumulate in CBs^10,24^, we checked for co-localization between coilin and the U4/U6 di-snRNP-specific protein Prp4k or the U5 snRNP-specific protein EFTUD2 in TFIP11-depleted cells. Both Prp4k and EFTUD2 were observed in coilin-positive CBs upon TFIP11 knockdown (Fig. 5h, i), suggesting that TFIP11 knockdown caused the retention of immature snRNPs stalled at intermediate assembly stages in CBs. In addition, these two proteins also accumulated in enlarged, irregularly shaped nuclear speckles (Supplementary Fig. 9), suggesting an imbalance in the flux of splicing factors into and out of these storage sites.

### TFIP11 regulates 2’-O-methylation of U6 snRNA

**Fig. 6:**
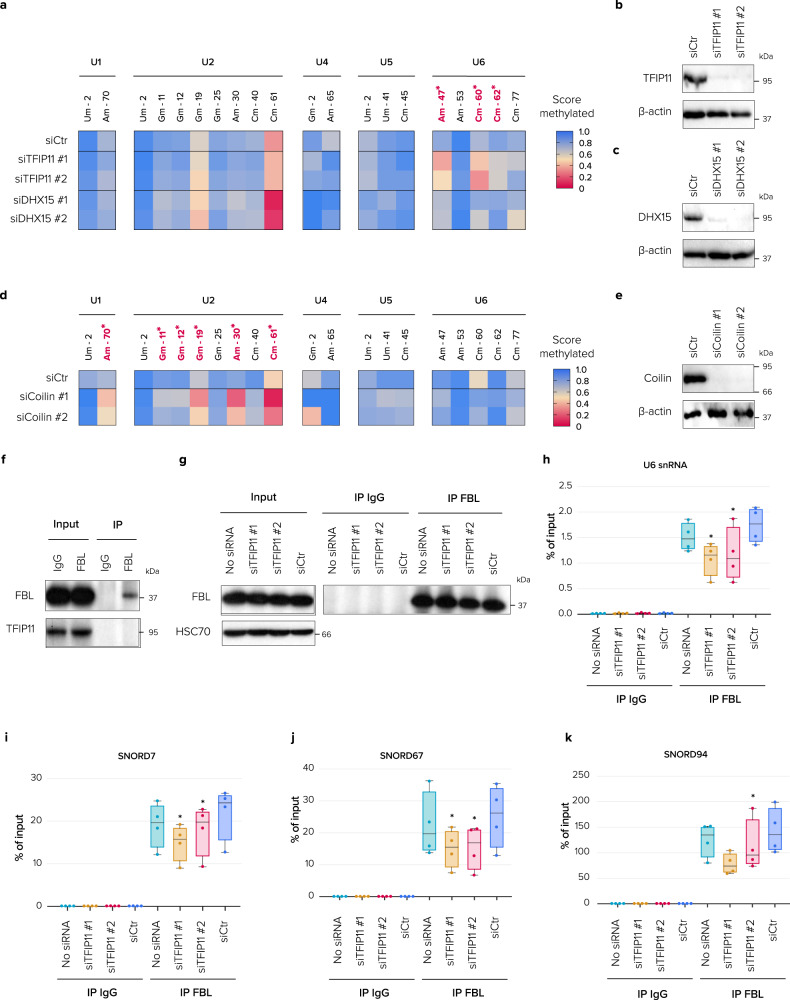
TFIP11 but not DHX15 is required for U6 snRNA 2’-O-methylation. **a**, **b** Identification of 2′-O-methylation on U snRNAs in HCT116 cells transfected for 72 h with control siRNA (siCtr), one of two different siRNAs against TFIP11 (siTFIP11 #1 and siTFIP11 #2) or one of two different siRNAs against DHX15 (siDHX15 #1 and siDHX15 #2). The heatmap displays the proportion of methylation for each modified position (column). Modified positions are highlighted as follows: asterisk = a position particularly sensitive to TFIP11 knockdown; red = hypomodification. **b**, **c** Protein extracts were analyzed by western blotting with antibodies against TFIP11 (**b**), DHX15 (**c**), and β-actin (loading control). **d** Identification of 2′-O-methylation on U snRNAs in HCT116 cells transfected for 72 h with control siRNA (siCtr) or one of two different siRNAs against coilin (siCoilin #1 and siCoilin #2). The modified positions are highlighted as follows: asterisk = a position particularly sensitive to coilin knockdown; red = hypomodification. **d** Protein extracts were analyzed by western blotting with antibodies against coilin and β-actin (loading control). **f** Immunoprecipitation (IP) of endogenous FBL from HeLa cell extracts followed by western blotting (WB) for the indicated proteins. **g** Western blotting for the indicated proteins after RNA IP (RIP) shows the IP of FBL in HeLa cells mock-transfected (No siRNA) or transfected with control siRNA (siCtr) or one of two different siRNAs against TFIP11 (siTFIP11 #1 and siTFIP11 #2). **h**–**k** Immunopurification of RNA-FBL complexes and RT-qPCR for U6 snRNA (overall *p* = 0.022, *p* = 0.032 for TFIP11#1, *p* = 0.038 for TFIP11#2, *n* = 4 independent experiments) (**h**) and its guide snoRNAs: SNORD7 (overall *p* = 0.019, *p* = 0.035 for siTFIP11#1, *p* = 0.029 for siTFIP11#2, *n* = 4 independent experiments) (**i**), SNORD67 (overall *p* = 0.019, *p* = 0.013 for siTFIP11#1, *p* = 0.011 for siTFIP11#2, *n* = 4 independent experiments) (**j**) and SNORD94 (overall *p* = 0.036, *p* = 0.074 for siTFIP11#1, *p* = 0.04 for siTFIP11#2, *n* = 4 independent experiments) (**k**) in HeLa cells transfected as in **g**. β-actin mRNA in IP beads served as an internal qPCR normalization reference. *p*-values were calculated using repeated measures ANOVA with post-hoc two-tailed paired *t*-tests. Only *p*-values < 0.05 (relative to siCtr) are displayed. Box limits = 25th to 75th percentiles; line = median; whiskers = min to max. Source data are provided as a Source Data file.

2’-O-methylation is one of the most common post-transcriptional modifications of U snRNAs and is required for their full activity. These modifications may play a role in modulating U snRNA- UsnRNA interactions, U snRNA-pre-mRNA interactions, and U snRNA-protein binding, consequently facilitating proper spliceosome assembly and activation^5^. Considering that TFIP11 binds to U snRNAs and also to sca/snoRNAs, and that it localizes to CBs and nucleoli where U snRNAs are modified (either by scaRNAs or snoRNAs, respectively), we next examined whether TFIP11 could regulate U snRNA 2’-O-methylation. To address this question we performed RiboMethSeq (RMS), a deep sequencing-based protocol allowing the systematic detection and quantification of RNA 2’-O-methylation^37,38^. Among U snRNAs, the 2′-O-methylation of U6 snRNA at positions A47, C60 and C62 was reduced to ~50% in TFIP11-depleted cells, though strikingly this was not the case upon depletion of DHX15 or coilin (Fig. 6a to e), indicating strong specificity of the methylation defect. A further indication of specificity is that 2′-O-methylation of the other U snRNAs was largely unchanged (Fig. 6a). Ribosomal RNA methylation remained largely unchanged upon TFIP11 knockdown but was distinctly affected following DHX15 depletion (Supplementary Fig. 10). These data indicate that DHX15 and TFIP11 independently control modifications of two distinct classes of RNA. This further corroborates the notion that TFIP11 and DHX15 function at least partially independently from one another despite the fact they interact to some extent (see Fig. 1a). Interestingly, a further RMS experiment demonstrated that coilin depletion did not alter the methylation level of U6 snRNA, but rather induced hypomethylation of U2 snRNA at positions G11, G12, G19, A30 and C61, and to a lesser extent U1 snRNA at position A70 (Fig. 6d, e). Importantly, a time course analysis revealed that the effect of coilin depletion on U1 and U2 modification was directly correlated to the level of coilin knockdown (Supplementary Fig. 11). In conclusion, these data indicate that, although TFIP11 and coilin physically interact and bind many of the same RNA targets, any functional relationship between these two proteins is unlikely involved in regulating 2’-O-methylation of U snRNAs.

To establish how TFIP11 contributes to the 2′-O-methylation of U6 snRNA, we first checked whether TFIP11 was associated with box C/D sca/snoRNP core proteins. Although TFIP11 interacts with box C/D sca/snoRNAs (Fig. 4), it did not co-immunoprecipitate with FBL, indicating that TFIP11 is not part of mature sca/snoRNPs (Fig. 6f). Since TFIP11 is not stably associated with box C/D snoRNP core proteins, we reasoned the protein might be involved in facilitating interaction between U6 snRNA and its modification guide snoRNAs, as was recently reported for nucleophosmin’s role in box C/D snoRNP-guided 2’-O methylation of pre-rRNA^39^. To test this idea, we performed RNA IP (RIP)-qPCR. Briefly, we affinity-purified FBL-associated RNAs (Fig. 6g), in the presence or absence of TFIP11, and tested for the presence of U6 snRNA and its modification guide snoRNAs by qPCR. This revealed that the binding of U6 snRNA to FBL was reduced approximately 30% upon TFIP11 knockdown (Fig. 6h). The level of association of FBL with SNORD7, SNORD67 and SNORD94, which modify U6 snRNA, was similarly reduced (Fig. 6i to k). However, the association of FBL with two snoRNA guides, SNORD56 and SNORD53, that target modification of 18 S and 28 S rRNA, was not affected upon TFIP11 depletion (Supplementary Fig. 12a, b). Together with our observation that TFIP11 displays a distinctive pattern of interaction with U6–methylating snoRNAs (see Supplementary Fig. 7), these results suggest that TFIP11 is important for the interaction of U6 snRNA with its snoRNA guides within FBL complexes, and that this facilitator role is crucial for efficient 2′-O-methylation of U6 snRNA.

### TFIP11 knockdown induces splicing defects

**Fig. 7:**
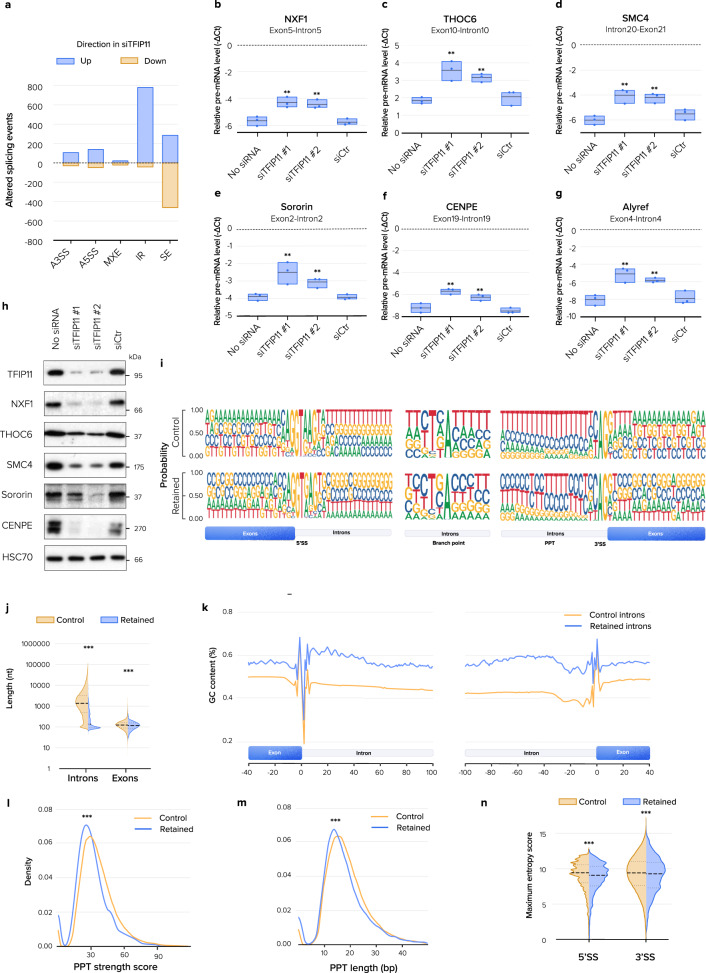
TFIP11 knockdown induces intron retention. **a** Number of differential alternative 5′ splice site (A5SS), alternative 3′ splice site (A3SS), mutually exclusive exon (MXE), retained intron (RI), and skipped exon (SE) events identified with rMATS in cells transfected for 72 h with one TFIP11 siRNA (siTFIP11 #1) compared to control siRNA (siCtr). **b**–**g** RT-qPCR shows increased intron retention in mRNA encoding NXF1 (DPSI by RNA-seq: 0.07; overall *p* = 8.4e-4, *p* = 0.0027 for siTFIP11#1, *p* = 0.0032 for siTFIP11#2), THOC6 (DPSI by RNA-seq: 0.14; overall *p* = 0.0015, *p* = 0.0049 for siTFIP11#1, *p* = 0.042 for siTFIP11#2), SMC4 (DPSI by RNA-seq: 0.01, overall *p* = 0.0017, *p* = 0.0054 for siTFIP11#1, *p* = 0.0041 for siTFIP11#2), sororin (DPSI by RNA-seq: 0.11; overall *p* = 0.0041, *p* = 0.0072 for siTFIP11#1, *p* = 0.0052 for siTFIP11#2), CENPE (DPSI by RNA-seq: 0.09; overall *p* = 3.79e-4, *p* = 0,001 for siTFIP11#1, *p* = 0.0086 for siTFIP11#2) and Alyref (DPSI by RNA-seq: 0.06; overall *p* = 0.0014, *p* = 0.0055 for siTFIP11#1, *p* = 0.0064 for siTFIP11#2) in cells transfected with one of two TFIP11 siRNAs (siTFIP11 #1 and siTFIP11 #2) compared to control siRNA (siCtr) for 72 h or mock-transfected cells (No siRNA). *n* = 3 independent experiments. *p*-values calculated using one-way ANOVA with Tukey post-hoc test. Only *p*-values < 0.05 (relative to siCtr) are displayed. Box limits = 25th to 75th percentiles; line = median; whiskers = min to max. **h** Western blotting in HeLa cells transfected as in **b** shows decreased abundance of indicated proteins whose mRNAs have increased IR. **i** Sequences at 5′ and 3′ splice sites (SS), branch points and polypyrimidine tracts (PPT) for introns with increased retention in cells transfected as in **a** (*n* = 2311 introns) and introns from all detected splicing events (*n* = 104,230 introns). **j** Length of introns and their flanking exons. Control and retained introns as in **i**. *p*-values calculated using Welch’s two-sample *t*-test. **k** GC content near 5′ and 3′ splice sites. Control and retained introns as in **i**. **l**–**m** Strength and length of PPT of introns. Control and retained introns as in **i**. *p*-values calculated using Welch’s two-sample *t*-test. **n** Strength of 5′ and 3′ splice sites (SS). Control and retained introns as in **i**. MaxEntScores ≥ 0 are displayed. *p*-values calculated using all values with Welch’s two-sample *t*-test. Source data are provided as a Source Data file.

Impaired snRNP assembly can prevent spliceosome activation and lead to splicing defects. To test the requirement of TFIP11 for pre-mRNA splicing competency at the transcriptome level, poly(A) + RNA from TFIP11-depleted cells was deep-sequenced. A total of 1937 alternative splicing events (ASE) were identified by rMATS in TFIP11-depleted cells, including alternative 3’ splice sites, alternative 5’ splice sites, mutually exclusive exons, skipped exons and intron retention (IR) (Fig. 7a). In particular, intron retention dramatically increased in TFIP11-depleted cells and IR was further examined with IRFinder. Affected introns were retained to different extents, with a median increase in retention of ~10% (Supplementary Fig. 13a). Quantitative reverse transcription PCR (RT-qPCR) experiments on selected IR events with different levels of retention confirmed the accumulation of retained introns (Fig. 7b to g). The level of corresponding protein was decreased in TFIP11-depleted cells, suggesting that IR is associated with lower protein levels (Fig. 7h). In contrast, DHX15 knockdown caused more exon skipping splicing events than intron retention (Supplementary Fig. 13b). Of note, < 10% of retained introns induced by TFIP11 knockdown were also induced in DHX15 depleted conditions (Supplementary Fig. 13c), and no IR on PCR-validated mRNAs was observed upon DHX15 depletion (Fig. 14d–i), further strengthening our conclusion that TFIP11 and DHX15 function independently. We did not observe a significant correlation between intron retention and gene expression level or change in expression level upon TFIP11 depletion (Supplementary Fig. 13j–k). Most genes with altered IR only showed increased retention of one intron. These retained introns were found distributed across the genes’ lengths, with introns further downstream in the transcript showing higher retention (Supplementary Fig. 13l–m). Deeper analysis revealed that retained introns differed from control introns in several specific *cis*-acting sequence features (Fig. 7i to n). Most strikingly, the retained introns were significantly shorter than average (median 126 nt vs 1585 nt). Their flanking exons were also statistically significantly shorter, though with a much smaller difference (median 106 nt vs. 114 nt) (Fig. 7j). Both the retained introns and their flanking exons had higher GC content (Fig. 7k). In the retained introns, the polypyrimidine tract tended to be weaker (Fig. 7l) and shorter (Fig. 7m), but no variations of the branch point sequence were observed (Fig. 7i). Both the 5’ splice site (5′-SS) and the 3’ splice site (3′-SS) encompassing the exon–intron and intron–exon boundaries were on average weaker than the corresponding sequences in control introns (Fig. 7i and n). Together these data suggest that some specific features of *cis*-regulatory elements in retained introns may influence intron retention upon TFIP11 knockdown.

### TFIP11 is required for mitosis and cell survival

**Fig. 8:**
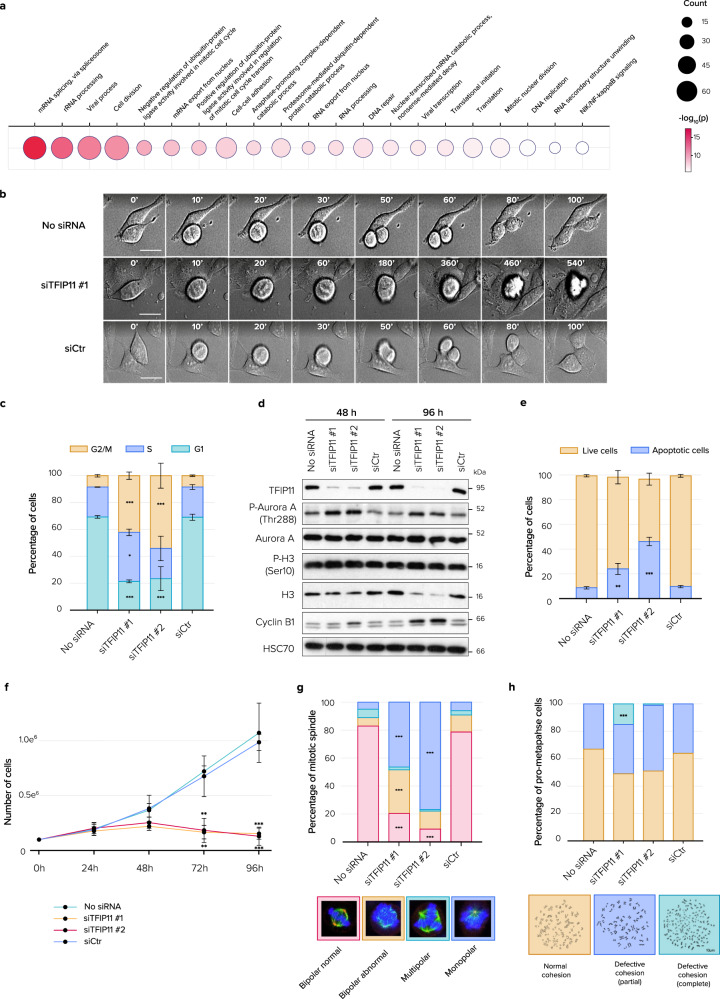
TFIP11 knockdown blocks cell cycle progression and induces apoptosis. **a** Enrichment of Gene Ontology (GO) terms in genes with increased IR upon TFIP11 depletion. Points are scaled by number of genes and colored by *p*-value. Enrichment analysis was performed and *p*-values calculated with a hypergeometric test with Benjamini–Hochberg correction using DAVID. **b** Live-imaging experiments of HeLa cells mock-transfected (No siRNA), transfected with TFIP11 siRNA (siTFIP11 #1) or control siRNA (siCtr) beginning 72 h post-transfection. Scale bar = 5 µm. **c** Cell cycle analysis of HeLa cells mock-transfected (No siRNA) or transfected with one of two different TFIP11 siRNAs (siTFIP11 #1 and siTFIP11 #2) or control siRNA (siCtr). *p*-values were calculated using one-way ANOVA with Tukey post-hoc test. G2/M: siTFIP11#1 *p* = 1.3e-04, G2/M: siTFIP11#2 *p* = 1.4e-05, S: siTFIP11#1 *p* = 0.030, G1: siTFIP11#1 *p* = 7.0e-06, G1: siTFIP11#2 *p* = 9.7e-06. Only *p*-values < 0.05 (relative to siCtr) are displayed. **d** Protein extracts from HeLa cells transfected as in **c** were analyzed by western blotting for the indicated mitotic proteins at the indicated timepoints. **e** Quantification of apoptotic cell death in HeLa cells transfected as in **c**. *p*-values were calculated using ANOVA with Tukey post-hoc test. Only *p*-values < 0.05 (relative to siCtr) are displayed. **f** Growth curve analysis of HeLa cells transfected as in **c**. *p*-values were calculated using one-way ANOVA with Tukey post-hoc test. Only *p*-values < 0.05 (relative to siCtr) are displayed. Data are presented as mean values +/– SD. **g** Proportion of normal, abnormal, monopolar, and multipolar spindles in mitotic HeLa cells transfected as in **c**. (No siRNA *n* = 96 / siTFIP11 #1 *n* = 89 / siTFIP11 #2 *n* = 136/ siCtr *n* = 118). *p*-values were calculated by Chi-square 2-sample test for equality of proportions with continuity correction and Bonferroni correction. **h** Quantification of the percentages of prometaphase phenotypes of HeLa cells transfected as in **c** (No siRNA *n* = 82/siTFIP11 #1 *n* = 56/siTFIP11 #2 *n* = 69/siCtr *n* = 91). Representative images of mitotic chromosome spreads observed in HeLa cells transfected as in **c**. *p*-values were calculated by Chi-square 2-sample test for equality of proportions with continuity correction and Bonferroni correction. Source data are provided as a Source Data file.

Gene Ontology (GO)-term analysis using DAVID was performed on genes with differential intron retention and showed that genes involved in mitosis and cell division were aberrantly spliced in TFIP11-depleted cells (Fig. 8a and Supplementary Data 2). This prompted us to investigate the cellular consequences of TFIP11 knockdown. Live-imaging experiments revealed aberrant mitotic progression upon TFIP11 knockdown and showed that prolonged mitotic arrest was often followed by apoptosis of the arrested cells (Fig. 8b). M phase arrest upon TFIP11 depletion was confirmed by cell cycle analysis (Fig. 8c and Supplementary Fig. 14a to c) as well as by western blotting against mitotic markers showing induction of phospho-Aurora A on Threonine 288, phospho-histone H3 on Serine 10 and cyclin B1 (Fig. 8d). Apoptotic cell death was also confirmed by Annexin V apoptosis assay (Fig. 8e). Apoptosis induction and cell cycle blockage upon TFIP11 knockdown translated into a significant decrease in cell number in vitro (Fig. 8f). In contrast, DHX15 knockdown did not induce the same cellular effects (Supplementary Fig. 15a, b). To further understand how TFIP11 knockdown compromises mitosis progression, we analyzed the mitotic profiles of TFIP11-depleted cells. Chromosome misalignments with failure of chromosomes to congress at the metaphase plate and defects in spindle polarity with appearance of monopolar spindles were frequently observed upon TFIP11 knockdown (Fig. 8g). The bipolar attachment of chromosomes to microtubules and the tension required to initiate anaphase depends on correct sister chromatid cohesion (SCC)^40^, a process especially sensitive to alterations in the splicing machinery^41^. In control cells, SCC was normal with mitotic chromosomes displaying the characteristic X shape. In contrast, TFIP11 knockdown led to partial or complete loss of SCC (Fig. 8h). Together these results indicate that altering TFIP11 expression level induces abnormal mitosis linked to defective SCC, leading to genomic instability and apoptosis.

## Discussion

Acting as a co-factor of the ATP-dependent DEAH-box RNA helicase DHX15, the G-patch protein TFIP11 was initially described in yeast as a splicing factor that catalyzes the disassembly of the lariat-spliceosome^12–15^. In contrast to these studies, we reveal DHX15-independent functions for TFIP11 in human cells and demonstrate that TFIP11 should be considered a key assembly factor, regulating the 2’-O-methylation of U6 snRNA, U4/U6.U5 tri-snRNP formation and spliceosome assembly. Indeed, RiboMethSeq analysis demonstrated that TFIP11 is crucial for the 2′-O-methylation of U6 snRNA, a process that is believed to occur in the nucleolus. Our observation that TFIP11 partially localizes to nucleoli, and the presence of a putative nucleolar localization signal (NoLS) at amino acids 240–243 (KKPK) located in the N-terminal IDP region of TFIP11 suggests that TFIP11 might be involved in the rapid and transient trafficking of U6 snRNA in and out of the nucleolus. Mechanistically, our data demonstrate that TFIP11 knockdown reduces the binding of U6 snRNA and its modification guide snoRNAs to FBL and alters U6 snRNA 2’-O-methylation. A related model was recently proposed in mouse germ cells in which LARP7 physically interacts with FBL and connects U6 snRNA with box C/D snoRNPs in order to promote U6 snRNA 2’-O-methylation^42,43^. However, we found that TFIP11 controls differential methylation of U6 snRNA by interacting with specific box C/D snoRNAs but not necessarily with FBL. This mechanism of action is more reminiscent of what has been observed for FRMP or nucleophosmin in their roles in rRNA modification^39,44^. The observation that TFIP11 is not stably associated with FBL suggests that TFIP11 is not a shared component of the snoRNP machinery involved in the modification of all U snRNAs but rather that TFIP11 might act as a specific accessory protein of the snoRNP that modifies U6 snRNA and become associated, transiently but functionally, with FBL only once it is bound to U6 snRNA.

The localization of TFIP11 in CBs as well its physical interaction with coilin and its association with shared RNA targets^27^ first suggested to us that the TFIP11-coilin complex could be a master regulator of Sm-class U snRNA modification in CBs. However, RiboMethSeq analysis demonstrated that TFIP11 and coilin have different roles in the modification of the Sm-class U1, U2, U4 and U5 snRNAs. While modifications of Sm-class U snRNAs are not sensitive to TFIP11 inhibition, 2′-O-methylation of U1 and U2 snRNAs is decreased in the absence of coilin, suggesting that TFIP11 and coilin are not functionally coupled to regulate post-translational modification of U snRNAs.

The association of TFIP11 with U4, U5 and U6 snRNAs and its stable interaction with both U4/U6 di-snRNP and U5 snRNP-specific proteins, along with the alteration of U4/U6.U5 tri-snRNP assembly upon TFIP11 depletion, support the notion that the localization of TFIP11 in CBs is required for the correct assembly and dynamics of U4/U6.U5 tri-snRNP particles. As suggested for other tri-snRNP assembly factors, the increased number of CBs observed upon TFIP11 depletion may compensate for tri-snRNP maturation defects^10,24^. These CBs exhibit structural features of CB but they are more heterogeneous in terms of size and morphology, as evidenced by immunofluorescence and TEM microscopy. The exact nature of these CBs is still obscure, and further work will be required to establish whether they are incompletely assembled CBs or fragmented bodies. Whether the maturation of Sm-class U snRNAs is conserved in these more heterogeneous CBs also deserves to be investigated. As the CBs remain positive for NOP58 after TFIP11 depletion, it is plausible that they are still functional and that U1, U2, U4 and U5 snRNA methylation still occurs there.

One key question concerns the exact contribution of TFIP11-dependent 2’-O-methylation of U6 snRNA in regulating the metabolism of the U4/U6.U5 tri-snRNP complex. There are eight methylation sites (A47, A53, G54, C60, C62, C63, A70, and C77) and three pseudouridylation sites (U31, U40, and U86) in U6 snRNA. A dynamic relationship between these post-transcriptional modifications and the structure and function of the U6 snRNA could be crucial for the mechanics of tri-snRNP assembly and disassembly. It is possible that hypomethylated A47, C60 and C62 nucleotides in U6 snRNA alter base-stacking and modify U6’s base-pairing interaction with U4 snRNA^7^. The absence of U6 snRNA modifications may also weaken the interaction with proteins important for the formation and/or stability of the tri-snRNP complex^45^. The U4/U6 snRNA duplex must be unwound for U6 snRNA to base pair with U2 snRNA and for the catalytically active spliceosome to form. This unwinding event is regulated by SNRNP200^46^. The continued presence of snRNP200 in the tri-snRNP as well as in higher spliceosomal intermediates (Fig. 5c, d) suggests that the U4/U6 di-snRNP structure in TFIP11-depleted cells still allows recognition by SNRNP200. However, its persistent presence accompanied by accumulation of U1 snRNA reflects inefficient U4/U6 unwinding and accumulation of stalled spliceosomes in a B-like conformation. Whether hypomethylated nucleotides in U6 snRNA hinder the intramolecular folding required for efficient unwinding^47^ by SNRNP200 deserves to be further investigated experimentally. Interestingly, Sidarovich et al. demonstrated that the cp028 compound stalls the spliceosome at an intermediate stage between B and B^act^ and this cp028-mediated conformation is associated with structural rearrangement of U6 snRNA^48^. This study further supports our hypothesis that alteration in U6 snRNA modification mediated by TFIP11 depletion is the main molecular mechanism responsible for the defect in spliceosome assembly. However, we cannot exclude that the physical presence of TFIP11 in the tri-snRNP and B complex, observed here and by others^49^, might also be essential to directly regulate dynamic protein-protein interactions in this complex. How TFIP11 knockdown or specific 2’-O-methylation of U6 snRNA globally change the stability, kinetics or dynamics of interactions within the spliceosome complex to dictate splicing outcomes deserves to be further studied by cryo-EM and proteomics. Whatever the changes in the conformation and composition of the spliceosome, our data demonstrate that TFIP11 knockdown affects splicing activity and results in widespread intron retention, demonstrating a key role for TFIP11 in normal splicing function. Interestingly, Dvinge et al. recently demonstrated that U6 snRNA knockdown also primarily induces intron retention^50^. As U6 snRNA is the core catalytic element of the spliceosome, alterations in the abundance of U6 snRNA might impede spliceosome catalytic activity, leading to intron retention. We speculate that the loss of U6 2’-O-methylation we observe upon TFIP11 depletion results in reduced U6 snRNA functionality, and therefore intron retention consequences that are similar to those of U6 snRNA knockdown.

One surprising observation stemming from our study is the DHX15-independent function of TFIP11. We initially assumed that, like in Saccharomyces cerevisiae, human TFIP11 was acting as a co-factor of human DHX15, and we expected to see the same molecular and cellular effects upon their respective knockdown. However, our findings showed that depletion of these proteins alters 2’-O-methylation of different RNA populations, differences in altered splicing events, and different cellular phenotypes. Thus, although TFIP11 and DHX15 proteins are present, at least to some extent, in the same protein complexes, it seems that the function of TFIP11 is not exclusively tied to regulation of DHX15 activity. Since several other G-patch proteins such as RBM5^51^, GPATCH2^52^ and PINX1^53^ can stimulate the helicase activity of DHX15, we suggest that the activation of DHX15 and its role in the activation of the spliceosome is mainly TFIP11-independent, and requires interaction and activation by another G-patch co-factor. In addition, it is noteworthy that RiboMethSeq analysis revealed that DHX15 knockdown reduced 2′-O-methylation of rRNAs in a TFIP11-independent manner, consistent with published works showing that the role of DHX15 in rRNA biogenesis depends on its interaction with other G-patch protein cofactors^16,53,54^.

At the cellular level, our data show that TFIP11 knockdown triggered abnormal mitosis and compromised cell cycle progression and cell survival. Mechanistically, TFIP11 knockdown induced premature loss of sister chromatid cohesion, thus preventing the stable attachment of microtubules and the bi-orientation of chromatids. Mis-splicing of mRNA encoding sororin (Fig. 7e), a cohesin-associated protein that stabilizes sister chromatids, is probably one of the mechanisms that links TFIP11 depletion with chromosome mis-segregation, as it was also observed upon inhibition of other splicing factors^41^. However, we cannot exclude that the M phase arrest and SSC defect might result from aberrant splicing in multiple mitotic genes such as SMC4^55^ or CENPE.

With their exceptional spatio-temporal heterogeneity and high conformational flexibility, intrinsically disordered proteins (IDPs) can function as “hubs” in complex protein-protein or protein-RNA interaction networks and can induce formation of membrane-less organelles such as Cajal Bodies or nucleoli by liquid–liquid phase separation (LLPS)^19^. The low complexity and intrinsically disordered regions (IDRs) in TFIP11 could be the molecular driver for its binding to multiple protein (coilin, DHX15, di-and tri-snRNP-specific proteins, etc.) and RNA (U snRNAs, snoRNAs, etc.) partners and for its role in the assembly and dynamics of the spliceosome complex. It is also tempting to hypothesize that the IDR of TFIP11 could interact with U snRNAs and/or sca/snoRNAs and then interact with coilin to contribute together to the structure, assembly, and function of phase-separated cellular compartments such as CBs. This might explain the disorganization of CB structures and the remarkable coalescence of coilin and SMN to the DFC of the nucleolus upon TFIP11 depletion. Interestingly, CK2 phosphorylation has been shown to play a role in the folding of IDPs^56^, and TFIP11 has been reported to be highly phosphorylated^57^, with three potential CK2-dependent phospho-sites (S59, S98 and S210) located in its N-terminal IDR. Whether these CK2-dependent phosphorylation sites are important for TFIP11’s LLPS behavior is an open question. Notably, targeting the folding repertoire of IDPs with small molecules represents a promising therapeutic strategy for many diseases. Successful small molecule inhibitors have been discovered and designed for several IDPs involved in cancers, such as c-Myc and EWS-Fli1^58^, indicating that IDPs are indeed druggable. As an IDP with a major role in spliceosome assembly, TFIP11 therefore holds important potential for therapeutic intervention in spliceosome-associated diseases.

## Methods

### Cell culture

HeLa (ATCC, #CCL-2), HCT116 (ATCC, #CCL-24), A549 (ATCC, #CCL-185) and MDA-MB-231 (ATCC, #HTB-26) cancer cell lines were maintained in Dulbecco’s Modified Eagle’s Medium (Lonza). U2OS cells (ATCC, #HTB-96) were maintained in McCoy’s 5A Medium. All media were supplemented with 10% heat-inactivated fetal bovine serum, and cells were incubated at 37 °C in the presence of 5% CO_2_. All cell lines were characterized by ATCC. All cells were routinely (every two weeks) examined for mycoplasma by MycoAlertTM Mycoplasma Detection Kit (Lonza, #LT07-118).

### siRNA transfection

siRNAs were synthesized by Eurogentec and were used at a final concentration of 40 nM. Cells were plated 24 h before transfection and then transfected at a confluence of approximately 50% using the calcium phosphate method. Mock-transfected cells (No siRNA) and cells transfected with an irrelevant siRNA targeting Gl3 luciferase (siCtr) were used as negative controls. Two different siRNAs targeting TFIP11 were used unless indicated. The sequences of the siRNAs are listed in Supplementary Table 1.

### Plasmid DNA transfection

Transient plasmid DNA transfection was carried out using Lipofectamine 2000 transfection reagent (Thermofisher, #11668030) according to manufacturer’s instructions. Plasmids encoding different constructs of TFIP11 cDNA in fusion with FLAG were generated by e-Zyvec. The plasmid encoding coilin cDNA with N-terminal OFPSpark / RFP tag was from Sinobiological.

### Total and nuclear protein extraction

For total protein extraction, adherent and floating cells were collected and lysed with a lysis buffer containing 1% SDS, 40 mM Tris–HCl (pH 7.5), 1 mM EDTA, complete EDTA-free protease inhibitor (Sigma-Aldrich, #11697498001) and phosphatase inhibitor (Sigma-Aldrich, #0490684500).

For nuclear extracts, cells were removed from dishes by trypsinization and washed twice with ice-cold PBS. Cells were resuspended in HMKE buffer (20 mM HEPES pH 7.2, 10 mM KCl, 5 mM MgCl_2_, 1 mM EDTA, and 250 mM sucrose) containing complete EDTA-free protease inhibitor, phosphatase inhibitor and digitonin (200 µg/ml). Cells were left on ice for 10 min and then centrifuged at 500 × *g* for 10 min at 4 °C. The supernatant (cytosol fraction) was carefully removed, and the pellet was washed twice in HMKE buffer without digitonin. To perform nuclear protein extraction, the pellet was solubilized in extraction buffer (0.1 M Tris–HCl pH 9, 100 mM NaCl, 5 mM KCl, 1 mM CaCl_2_, 0.5 mM MgCl_2_, 0.5% NP-40 supplemented with RNAsin (Promega, #N2115), complete EDTA-free protease inhibitor and phosphatase inhibitor) for 30 min on ice. The samples were then centrifuged at 15,000 × *g* for 10 min at 4 °C and the supernatant (nuclear fraction) was carefully removed. Fractionation was verified by western blotting using an antibody to Mek2 (cytoplasmic marker) and an antibody to Lamin A/C (nuclear marker).

### Western blotting (WB) analysis

Proteins were quantified with the BCA protein assay kit (Pierce, #23225) and separated by sodium dodecyl sulphate–polyacrylamide gel electrophoresis (SDS–PAGE) to be transferred onto a PVDF (Polyvinylidene fluoride) membrane. After blocking of non-specific binding sites, proteins were detected with primary antibody (Supplementary Table 2) and HRP-conjugated secondary antibody. Finally, membranes were developed using chemiluminescence detection. HSC70 or β–actin antibody was used as loading control.

### Glycerol-gradient (10–30%) fractionation

Nuclear extracts (300 µg) were diluted in 400 µl of IP150 buffer (HEPES pH 7.9, 150 mM NaCl, 1.5 mM MgCl_2_, 0.5 mM DTT, supplemented with RNasin (Promega, N2115) and complete EDTA-free protease inhibitor (Sigma-Aldrich, #11697498001) and phosphatase inhibitor (Sigma-Aldrich, #04906845001) and then loaded onto a linear 4 ml 10–30% (v/v) glycerol-gradient prepared with IP150 buffer. The samples were then centrifuged at 92,500 × *g* for 16 h at 4 °C in a Beckman SW60Ti rotor. Twenty-four fractions (170 µl) were collected from top to bottom using a Biocomp piston gradient fractionator (Biocomp). For each fraction, 50 µl were denatured with Laemmeli 6x and analyzed by western blotting. One-hundred microliters of each fraction were subjected to Tripure RNA extraction (Ambion, TRI reagent #AM9738). RNA was precipitated and washed in the presence of glycoblue (Invitrogen, #AM9515). Total RNA was separated on a denaturing acrylamide gel (8% acrylamide/50% urea/1x TBE), transferred to a nylon membrane (GE HealthCare, #RPN203B), and processed for northern blotting. Membranes were hybridized with a cocktail of radioactively labeled oligonucleotide probes specific to U snRNAs U1, U2, U4, U5, and U6. Each probe was used in equimolar amounts, except for U1, for which only half of the probe was used. The signal was captured on Fuji plates with a Fuji FLA-700 phosphorimager v1.1 and Multi Gauge software v3.1 (set to default parameters). The probe sequences are listed in Supplementary Table 3.

### Co-immunoprecipitation (Co-IP)

HeLa cells were washed with PBS, lysed with RIPA buffer and then incubated at 4 °C for 30 min with rotation. After sonication, the supernatant was clarified by centrifugation at 5000 × *g* for 10 min at 4 °C. Protein extracts were then diluted with dilution buffer (50 mM Tris–HCl pH 7.5, 150 mM NaCl). The final concentrations of Triton X-100 and SDS in the immunoprecipitation reaction were 0.1% and 0.01%, respectively. Proteins were then incubated with the indicated antibodies (Supplementary Table 4) conjugated with agarose beads (Santa Cruz Biotechnology, #sc2003) at 4 °C overnight. IgGs of the corresponding species were used as negative controls. The beads were washed four times with washing buffer (50 mM Tris–HCl pH 7.5, 150 mM NaCl, 0.1% Triton X-100), resuspended in 2x Laemmli buffer, boiled for 10 min and centrifuged at 15,000 × *g* for 5 min. Purified proteins were resolved by SDS–PAGE and subjected to western blotting analysis.

For Fig. 5a, c HeLa cells were washed with ice-cold PBS, lysed with lysis buffer (Tris–HCl 0.2 M, pH 7.5, NaCl 500 mM, EDTA 5 mM, EGTA 5 mM, Triton X-100 1%, complete EDTA-free protease inhibitor (Sigma-Aldrich, #11697498001) and phosphatase inhibitor (Sigma-Aldrich, #04906845001). Collected cells were sonicated and then incubated on ice for 30 min. The supernatant was clarified by centrifugation at 5000 × *g* for 10 min at 4 °C. After quantification, protein extracts were diluted to equal concentrations and then pre-cleared with protein A/G agarose beads (Santa Cruz Biotechnology, #sc-2003). After centrifugation at 5000 × *g* for 5 min at 4 °C, the supernatants were incubated on the rotor overnight at 4 °C with the indicated antibodies. IgGs of the corresponding species were used as negative controls. A/G beads were then added to the lysates for 2 h at 4°. After centrifugation (600 × *g* for 10 min at 4 °C), beads were collected and washed four times with lysis buffer, resuspended in 2X Laemmli buffer, boiled for 10 min and centrifuged at 5000 × *g* for 5 min. Purified proteins were resolved by SDS–PAGE and subjected to western blotting analysis. For the input lanes, 6% of the amount of total protein used in each IP condition (650 µg) was loaded.

### Immunofluorescence (IF)

HeLa cells were seeded on coverslips in 24-well plates. Cells were then rinsed with PBS, permeabilized with CSK buffer (300 mM sucrose, 3 mM MgCl_2_, 10 mM PIPES pH 7.0, 100 mM NaCl, 0.25% Triton X-100) and fixed with cold methanol (80)/acetone (20) for 10 min at −20 °C. After several washes, the coverslips were blocked in PBS with 1% BSA (PBS-BSA) for 30 min at room temperature and incubated with primary antibodies (Supplementary Table 5) diluted in PBS-BSA for 1 h at room temperature. The coverslips were then washed and incubated with appropriate AlexaFluor-conjugated secondary antibodies (Life Technologies) diluted in PBS-BSA at 1/2000. Nuclear cells were counterstained with DAPI (Life Technologies, #D1306) for 10 min at room temperature in the dark. The coverslips were mounted onto microscope slides and images were obtained with a Leica TCS SP5 laser scanning confocal microscope and visualized and analyzed using Imaris v8.

### In situ proximity ligation assay (iPLA)

HeLa cells were cultured on coverslips then washed with cold PBS and fixed with cold methanol for 20 min at −20 °C. Coverslips were washed again 4 times with cold PBS. All incubations were performed in a humidity chamber. Blocking was done with 0.1% tween-TBS with 1% BSA for 1 h at 37 °C. Incubations with primary antibodies were performed overnight at 4 °C. Cells were washed three times for 3 min with 0.1% tween-TBS with gentle agitation. Incubations with PLA Probe Anti-Mouse PLUS (Sigma-Aldrich, #DUO92001) and PLA Probe Anti-Rabbit MINUS (Sigma-Aldrich, #DUO92005) were performed for 2 h at 37 °C. Next, coverslips were washed three times for 3 min in T-PBS buffer under gentle shaking and then incubated with DNA ligase previously diluted in ligation buffer for 30 min at 37 °C according to manufacturer’s instructions. Coverslips were washed three times for 3 min in T-PBS buffer under gentle shaking and incubated with a DNA polymerase previously diluted in amplification buffer for 90 min at 37 °C. Finally, coverslips were rinsed for 10 min in the presence of DAPI under gentle shaking and then washed 2 min with 2X and then 0.02X SCC buffer (30 mM sodium citrate, 300 mM sodium chloride). Coverslips were mounted in Fluoromount Aqueous Mounting Medium (Sigma-Aldrich, #F4680) and analyzed using a Zeiss LSM confocal microscope.

### Transmission electron microscopy (TEM)

HeLa cells were fixed in 2.5% glutaraldehyde (diluted in Sorensen’s buffer: 0.1 M Na_2_HPO_4_/NaH_2_PO_4_ buffer, pH 7.4) for 1 h at 4 °C and postfixed in 2% OsO_4_ (diluted in 0.1 M Sorensen’s Buffer). After dehydration in graded ethanol, samples were embedded in Epon resin (Sigma-Aldrich, #45359). Ultrathin sections obtained with a Reichert Ultracut S ultramicrotome (Reichert Technologies) were contrasted with 2% uranyl acetate and 4% lead citrate. Observations were made with a Jeol JEM-1400 transmission electron microscope at 80 kV (Jeol).

For immunolabeling, U2OS cells were fixed in 4% formaldehyde/0.1% glutaraldehyde (diluted in 0.1 M Sorensen’s buffer) for 1 h, rinsed in buffer and dehydrated in graded ethanol baths. After dehydration, samples were infiltrated with increasing concentrations of Lowicryl K4M resin. Resin was further polymerized under ultraviolet light. Ultrathin sections were washed in saline phosphate buffer (0.1 M PBS, 0.14 M NaCl, 6 mM Na_2_HPO_4_, 4 mM KH_2_PO_4_, pH 7.2) then blocked for 30 min in PBS-BSA (1%, pH 7.2) supplemented with normal goat serum (1/30 dilution). Sections were washed in PBS-BSA (0.2%, pH 7.2) and incubated with rabbit anti-TFIP11 (Bethyl Laboratories, # A302-549A), 1/5 dilution in PBS-BSA (0.2%, supplemented with normal goat serum 1/50) for 3 h. After three washes in PBS-BSA (1%, pH 7.2) and an additional wash in PBS-BSA (0.2%, pH 8.2), sections were incubated with secondary antibody (anti-rabbit coupled with 10 nm gold particles (Aurion, #810.011) diluted 1/40 in PBS-BSA 0.2%, pH 8.2) for 1 h. Finally, sections were washed four times in PBS-BSA (0.2%, pH 8.2) and four times in deionized water, stained with uranyl acetate and lead citrate and allowed to dry. For negative controls, the sample was incubated with secondary antibody only (omitting the primary antibody step). For ultrastructural analyses, random fields of cells were examined under a Jeol TEM JEM-1400 Transmission Electron Microscope at 80 kV, and random fields were photographed using an 11-megapixel camera system (Quemesa, Olympus). Morphometric measurements were performed with iTEM v5.2 (Olympus, Tokyo, Japan) and analyzed using Image J v1.52a program.

### Live-cell imaging

After transfection, HeLa cells were plated in a cell culture dish with four individual compartments. Live-imaging for 24 h at a time lapse of 5 min was performed with a Nikon A1R microscope. Image J v1.52a program was used for image analysis.

### Cell cycle analysis

The relative percentage of cells in each stage of the cell cycle was analyzed by labeling nuclei with propidium iodide (PI) followed by flow cytometry analysis with FACS Calibur II^™^ and ModFit LT^™^ v3.2.1 program.

### Apoptosis analysis

Apoptosis was measured with the FITC-Annexin V apoptosis Detection Kit I (BD Biosciences, #556547) according to manufacturer’s instructions. Flow cytometry was performed and data were analyzed with CellQuest software v3.2 (BD Biosciences). Annexin V^+^/PI^-^ and annexin V^+^/PI^+^ cells were considered apoptotic.

### Growth curve analysis

After transfection, HeLa cells were trypsinized and viable cells were plated at a density of 100,000 cells per well in a six-well plate. Cell counting was carried out every 24 h for 4 days, and the medium was replaced every 2 days.

### Chromosome spreads

HeLa cells were treated with 0.1 µg/ml of colcemid for 16 h. Adherent and floating cells were collected, pelleted and resuspended in 10 ml heated hypotonic solution (8 g/l Na citrate, 0.075 M KCl, H_2_O, 1:1:1) at 37 °C for 20 min. Cells were then fixed with fresh fixation solution (absolute ethanol and acetic acid, 3:1). After several washes with fixation solution, cells were spread onto clean slides by dropping the cell suspension and then let to dry. Spreads were stained with Giemsa stain.

### Quantitative reverse transcription PCR (RT-qPCR)

Total RNA of HeLa cells was extracted using the NucleoSpin RNA kit (Macherey-Nagel, #740955) and measured by Nanodrop. Reverse transcription was performed with the RevertAid H minus first strand cDNA synthesis kit (ThermoFisher Scientific, #K163) according to the manufacturer’s instructions. Quantitative PCR was performed with a LightCycler 480 instrument (Roche, #04707516001) with β-actin as control. To detect retained introns (mRNA + IR), primers recognizing exon–intron junctions were used. Primers complementary to one exon were used for total RNA (mRNA + IR and mRNA). The relative abundance of pre-mRNAs with retained introns was determined as follows: –ΔCt = –(Ct_mRNA+IR_ – Ct_total RNA_). All measurements were realized in triplicate. Primer sequences are shown in Supplementary Table 6.

### iCLIP-Seq

HeLa cells were exposed three times to UV light at 400 mJ/cm^2^ for cross-linking and harvested with cold PBS. Cells were lysed in 500 µl of extraction buffer, incubated 5 min at 37 °C and centrifuged at 135 × *g* for 15 min at 4 °C. The supernatant was incubated with RNase T1 for 5 min at 37 °C then incubated with Dynabeads protein G (Life Technologies, #10003D) coupled with rabbit anti-TFIP11 antibody (Bethyl Laboratories, #A302-549A) for 2 h at 4 °C with rotation and washed five times with wash buffer (50 mM Tris–HCl pH 7.4, 1 M NaCl, 1% NP-40, 0.1% SDS, 0.5% DOC). Library construction was performed as described in Huppertz et al^.59^. After dephosphorylation of RNA 3’ ends, RNAs bound to TFIP11 were ligated on beads with P^32^-labeled L3A-linker. After elution, the RNA-protein complexes were separated by SDS–PAGE, transferred to a nitrocellulose membrane (Sigma, Protran BA85 #WHA10402506) and visualized by autoradiography. TFIP11-RNA complexes were isolated from the membrane and proteins were digested with proteinase K. RNA was eluted with phenol-chloroform and ethanol-precipitated. RNAs were size-selected on 6% acrylamide/urea gel and reverse transcribed with RCLIP primers targeting the L3 linker. cDNAs were then purified on a 6% acrylamide/urea gel, circularized with Circligase II (Epicentre, #CL9021), re-linearized with BamH1 and amplified by PCR to produce the sequencing library.

Library quality was assessed using a Bioanalyzer 2100 HS DNA assay and quantified by qPCR. Library sequencing was performed on 4 lanes of a Nextseq 550 (Illumina) (NextSeq 500/550 HM kit v2.5 75 cycles) to yield 92 nt long raw sequences. Fastq files were processed using the iCount v2.0.0 suite (https://hub.docker.com/r/tomazc/icount/). Briefly, data were demultiplexed based on barcode (NNNGGTTNN, NNNTTGTNN, NNNCAATNN or NNNGGCGNN) and adapters (AGATCGGAAGAGCGGTTCAGCAGGAATGCCGAGACCGATCTCGTATGCCGTCTTCTGCTTG) were processed. All reads originating from the same libraries were pooled and mapped to the hg19 genome using the version of STAR included with iCount v2.0.0. Unique cDNA were counted and significant peaks called using the iCount v2.0.0 peaks function.

To determine density of iCLIP tag mapping to different regions of snoRNAs, snoReport 2.0^60^ was used to determine locations of secondary structures based on primary transcript sequence. Box C/D snoRNAs were divided into stem, 5’ half of loop and 3’ half of loop regions. Box H/ACA snoRNAs were divided into 3’ stemloop, H box and 5’ stemloop regions. Tags mapping to each region were counted and divided by the total number of tags mapping to the snoRNA. Tag mapping locations were analyzed with R packages including dplyr v1.0.2; ggplot v2 3.3.2; tidyr v1.1.2; rtracklayer v1.34.2; biomaRt v2.30.0 and SuperExactTest. v1.0.7.

### RIP-PCR

HeLa cells were UV-crosslinked and lysed with 500 µl of RIPA buffer supplemented with RNasin (Promega, #N2115) and turboDNase (Invitrogen, #AM2238). After centrifugation, 10% of the supernatant was retained as the input sample and the same amount of protein for each condition was incubated overnight with agarose beads coupled with anti-FBL antibody (Bethyl Laboratories, # A303-891A) or rabbit IgG at 4 °C. Beads were then washed six times with RIPA buffer. Input and beads were incubated with proteinase K to digest protein. RNA in supernatants was extracted using the trizol/chloroform method and eluted in 20 µl of RNase-free water. For quantitative analysis of RNAs bound by FBL, the percentage of input was calculated for each condition as: 2^((Ct input – log2(10)) – Ct IP)^ x 100 (10 is the dilution factor of input used for quantitative analysis).

### RNA-seq

For mRNA-seq from HeLa cells, libraries were prepared with the Illumina TruSeq stranded mRNA protocol and 2 × 76-bp sequencing was performed on an Illumina NextSeq 500 instrument. RNA-seq read quality was evaluated using FastQC v0.11.3. Reads containing adapter sequences and/or low-quality ends were identified and trimmed using bbduk v35.59. The processed reads were mapped to the human genome (hg19) using STAR v2.5.2b. PCR duplicates were removed from aligned files using Picard tools v1.139, gene expression was quantified using featureCounts v1.4.6-p5 and GENCODE v.26 annotation and differential expression analysis was performed using edgeR v3.14.0. Differential intron-retention analysis was performed using IRFinder v1.2.3 using the audic-claverie-test as a test statistic. Features with a test statistic less than 0.01 were considered to be significantly different in terms of intron retention. Other alternative splicing events were assessed with rMATS v3.3.2.

Retained and non-retained introns and their flanking exons were analyzed using the Integrative Genomics Viewer (IGV) v2.5.2, BedTools v2.25.0 and R 3.3.1 with packages including dplyr v1.0.2, tidyr v1.1.2, biomaRt v2.30.0, ggplot2 v3.3.2, ggseqlogo v0.1, stringr v1.4.0 and Biostrings v2.42.1. Intron and exon characteristics (sequences, rank in transcript, etc.) were obtained from Ensembl 75 using biomaRt v2.30.0. When applicable, principal isoforms were selected using APPRIS 2017_10.v25 or the longest isoform. Splice site strength was assessed with MaxEntScan 2018-10-22. Predicted splice branch point locations were obtained from Signal et al^.61^. Polypyrimidine tract lengths and scores were assessed with BPfinder 2016. GO-term analysis was performed on genes with differential intron retention using DAVID v6.8^62^.

### Small non-coding RNA-seq

For small non-coding RNA-seq from HeLa cells, libraries were prepared using the Clontech SMARTer smRNA-Seq Kit for Illumina protocol. To enrich for transcripts in the snoRNA size range, size selection was performed with magnetic beads. 1 ×100-bp sequencing was performed on an Illumina NextSeq 500 instrument. Adapters, poly(A) tails, and 3’ bases were removed from RNA-seq reads with Cutadapt as per Clontech recommendations. RNA-seq read quality was checked using FastQC. Transcript expression (GENCODE v.26 annotation) was quantified using Salmon v0.8.2, which includes information from reads that map to multiple locations in the genome/transcriptome (including some reads arising from sn/snoRNA genes), and summed to gene level using tximport v1.2.0. Differential expression was assessed using DESeq2 v1.14.1. Genes for which adjusted *p*-value < 0.1 were considered to be differentially expressed. Additionally, reads were mapped to the human genome (hg19) using STAR v2.5.2b and coverage visualized using the Integrative Genomics Viewer (IGV) v2.5.2.

### RiboMethSeq (RMS)

For quantitative detection of sites of 2’-O ribose methylation, RiboMethSeq was performed on total RNA extracted from HCT116 cells mock-transfected (No siRNA) or transfected for 3 days with one of two siRNAs targeting mRNAs encoding TFIP11, DHX15 or coilin or with an irrelevant siRNA (siCtr). Two independent experiments were performed. RiboMethSeq libraries were prepared using 150 ng total RNA^38^. The samples were sequenced on an Illumina NovaSeq 6000 device as a paired-end run (100 nt read length).

For the analysis, adapter sequences were removed using Trimmomatic (0.36; LEADING:30 TRAILING:30 SLIDINGWINDOW:4:15 MINLEN:17 AVGQUAL:30) and read quality was assessed using FastQC v0.11. Reads in the forward direction were mapped to an artificial genome containing ribosomal RNA sequences using bowtie2 v2.4. Mapped reads were analyzed using samtools v1.9, snakemake-minimal v5.24.1 and the R package RNAmodR.RiboMethSeq v1.6.0 and the Score C/RiboMethScore were used as a measurement for 2’-O-methylation. For the analysis of methylation levels on positions known to be methylated, data from the snoRNAdb 2016 was used^63^ and updated to newer rRNA sequence coordinates based on NCBI accession NR_046235.3 available from the ‘EpiTxDb‘ package v1.3.3. Only the reads shorter than 90 nt were used for the analysis based on the length distribution of reads after adapter trimming. Only bases with sufficient read coverage for analysis were displayed. Summary information was obtained using multiqc v1.9.

### Investigation of intrinsic disorder properties

Three algorithms were used: MFDp2 v2.0, PrDOS v2.0 and DISOPRED v3.1. DISOPRED and PrDOS are machine learning predictors based on amino acid composition and/or on structural templates. MFDp2 is a multilayered fusion-based predictor or a meta-predictor, based on estimated disorder probabilities from other predictors. The percentage of order-promoting residues (OPR: Trp, Cys, Tyr, Ile, Phe, Val, Asn, and Leu), disorder-promoting residues (DPR: Arg, Pro, Gln, Gly, Glu, Ser, Ala, and Lys) and non-promoting order/disorder residues (NPR: Asp, Met, Thr and His) was calculated from the sequence of TFIP11, known IDPs (α synuclein, osteopontin, emerin) and known well-ordered proteins (lysozyme C, BSA). A Charge-Hydropathy (CH) plot was set up from the PONDR^®^v.2007 algorithm that distinguishes ordered and disordered proteins based only on net charge and hydropathy, introduced by Uversky et al.^19^. This plot compared the absolute, mean net charge— neglecting histidine—and the mean, scaled Kyte-Doolittle hydropathy^64^. The radius of gyration (Rg) was straightforwardly calculated from an atomistic model built by the I-Tasser algorithm^65^. The approach proposed by Tomasso et al. was used for the hydrodynamic radius (Rh) calculation^66^.

### Figures

Figure panels were assembled and final figures produced using Microsoft Powerpoint v14.7.2, v14.7.7 and v16.53; Adobe Photoshop.v13.0; Adobe Illustrator CC v2021 and GraphPad Prism v2.2.0.

### Statistics and reproducibility

Unless otherwise indicated, data are expressed as mean ± SD. Statistical tests were performed in R3.3.1 and are indicated in each figure legend. *p*-values less than 0.05 were considered statistically significant. *p < 0.05; **p < 0.01; ***p < 0.001 in figures.

Each immunoprecipitation experiment was independently performed three times; representative western blots are shown in Fig. 1a; 2b, c, f, h; 5a–c and 6f, g. Each iPLA experiment was independently performed three times; representative micrographs are shown in Figs. 1b and 2d.

Each microscopy experiment, including both EM with immunogold labeling and immunofluorescence, was independently performed at least three times on separately treated samples. Representative images are shown in Fig. 1d–I, o–p; 2a; 3a, d–h; 5h, i and 8g and Supplementary Fig. S1, S2, S4c, S5a, and S9.

Glycerol-gradient fractionations were independently performed three times. Nuclear extracts from TFIP11 siRNA or control siRNA (siCtr)-transfected cells were separated in parallel on a glycerol gradient. A representative western blot for relevant nuclear proteins is shown in Fig. 5g. One part of each fraction collected from the gradient was subjected to RNA extraction, while the second part was used for protein extraction. Northern and western blots for each experimental condition (transfections with TFIP11 siRNA or control siRNA (siCtr)) were entirely processed in parallel (migration, transfer and revelation). Representative northern and western blots from one experiment are shown in Fig. 5d, e.

For RNA-seq experiments in Fig. 4i–k and 7a, i–n and Supplementary fig. 13a–c, j–m, transfections with TFIP11 siRNA (siTFIP11#1), DHX15 siRNA (siDHX15 #1) or control siRNA (siCtr) were independently performed three times. RNA-sequencing analysis was done on the three independent RNA samples from the two experimental conditions.

For RiboMethSeq, transfections with TFIP11 siRNA (siTFIP11#1 or siTFIP11#2), Coilin siRNA (siCoilin #1 or siCoilin #2), DHX15 siRNA (siDHX15 #1 or siDHX15 #2), control siRNA (siCtr) were performed twice each. Representative western blots from one experiment are shown in Fig. 6b, c, e and Supplementary Fig. S11b.

For RIP-PCR experiments in Fig. 6h–k, transfections with TFIP11 siRNA (siTFIP11#1 or siTFIP11#2) or control siRNA (siCtr) or mock transfections were independently performed four times. RNA associated with fibrillarin in each experimental condition from the four independent experiments was analyzed by RT-qPCR.

For RT-qPCR analysis in Fig. 7b–g, transfections with TFIP11 siRNA (siTFIP11#1 or siTFIP11#2) or control siRNA (siCtr) or mock transfections were independently performed three times. The three independent RNA samples from the two experimental conditions were then analyzed by RT-qPCR using specific primers.

Transfections with TFIP11 siRNA (siTFIP11#1 or siTFIP11#2), Coilin siRNA (siCoilin), control siRNA (siCtr) or mock transfections (no siRNA) followed by western blots of relevant proteins were independently performed 3 times. Representative western blots from one experiment are shown in Figs. 7h and 8d and Supplementary Figs. S3, S4b, S5b and S8.

Transfections with TFIP11 siRNA (siTFIP11#1) or control siRNA (siCtr) or mock transfections followed by live-cell imaging were independently performed 2 times; representative images from one experiment are shown in Fig. 8b.

Transfections with TFIP11 siRNA (siTFIP11#1 or siTFIP11#2), DHX15 siRNA (siDHX15 #1 or siDHX15 #2), control siRNA (siCtr), or mock transfections (no siRNA) in various cell lines followed by cell cycle analysis were independently performed three times.. Representative western blots are shown in Supplementary Figs. S14 and S15.

### Reporting summary

Further information on research design is available in the Nature Research Reporting Summary linked to this article.

## Supplementary information

Supplementary Information

Description of Additional Supplementary Files

Supplementary Data 1

Supplementary Data 2

Reporting Summary

## Data Availability

The data supporting the findings of this study are available from the corresponding authors upon reasonable request. Sequencing data (RNA-Seq, RiboMethSeq, and iCLIP) generated in this study have been deposited in the NCBI GEO database under accession code GSE156390. Source data for the figures and supplementary figures are provided as a Source Data file. Source data are provided with this paper.

## Source data

Source Data
