# Supplementary material for: DHX15-independent roles for TFIP11 in U6 snRNA modification, U4/U6.U5 tri-snRNP assembly and pre-mRNA splicing fidelity
Source: Nat Commun. 2021 Nov 17;12:6648. doi: 10.1038/s41467-021-26932-2 (PMC8599867; doi:10.1038/s41467-021-26932-2)
Supplement: Supplementary file 1 — Supplementary Information [file 41467_2021_26932_MOESM1_ESM.pdf]

Supplementary Information for

DHX15-independent roles for TFIP11 in U6 snRNA modification, U4/U6.U5 tri-snRNP assembly and pre-mRNA splicing fidelity

Supplementary Figure 1

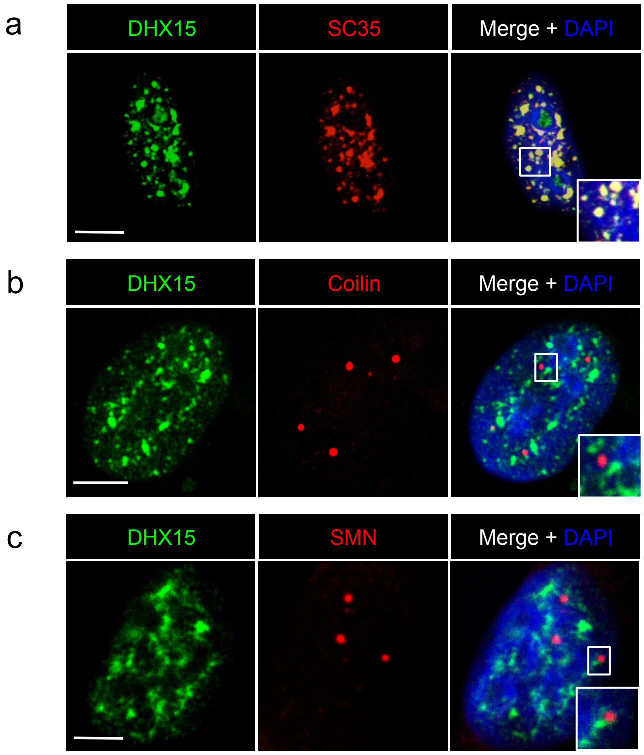

DHX15 co-staining with SC35, coilin and SMN proteins. HeLa cells were co-stained with anti-DHX15 antibody (in green) and antibodies against SC35 (a), coilin (b) and SMN (c) (in red). Individual channels, merged channels with nuclear staining (in blue) and magnification of boxed regions are shown. Scale bar = 5 µm.

Supplementary Figure 2

TFIP11 immunogold labelling

a

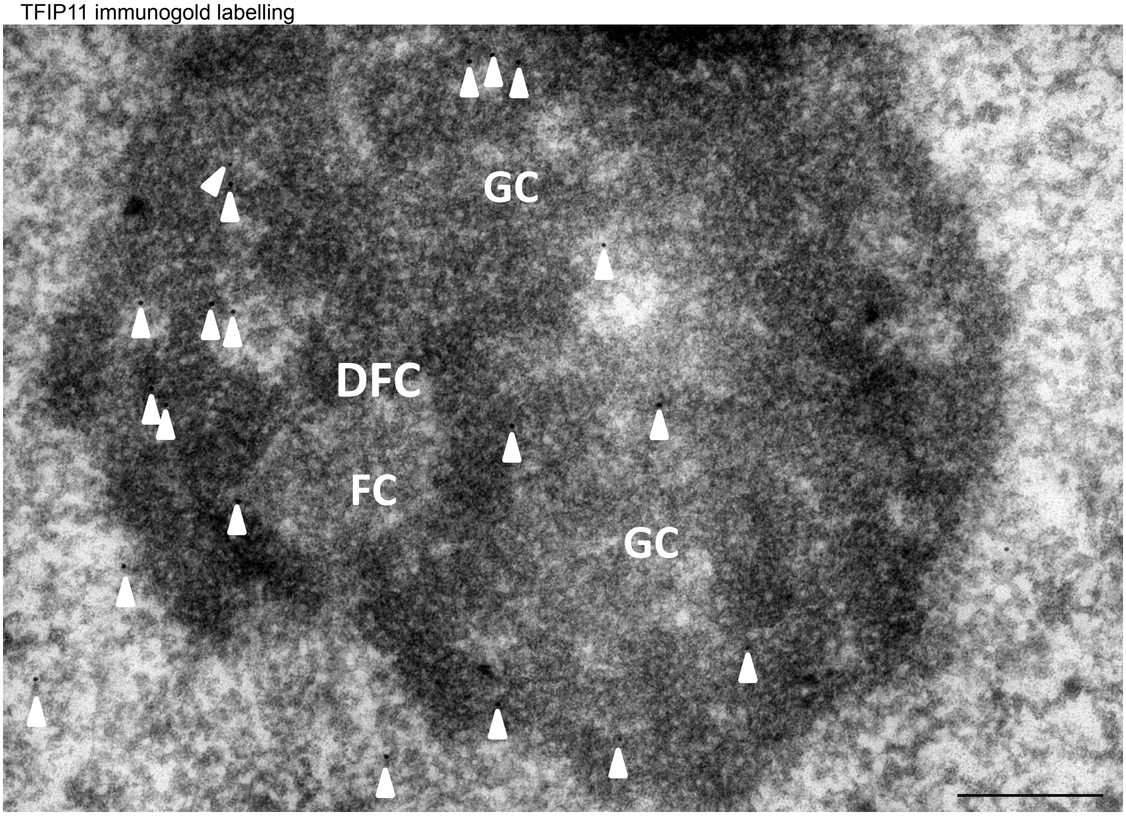

Negative Control (without Primary Antibody)

b

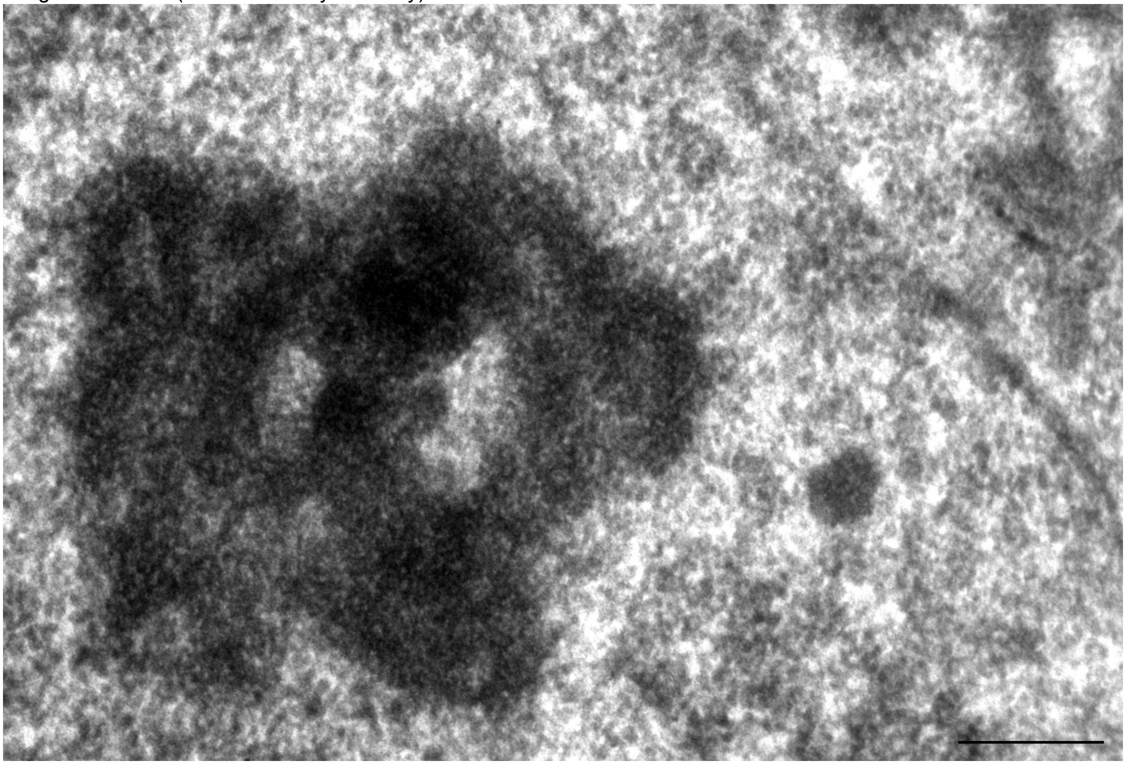

Distribution of endogenous TFIP11 in U2OS cells detected by immunogold/electron microscopy (EM). (a) Immuno-EM of sections incubated with primary antibody against TFIP11. White arrows delineate the accumulation of gold particles labeling endogenous TFIP11 in the three compartments of the nucleolus: the dense fibrillar center (DFC), the fibrillar center (FC) and the granular component (GC). TFIP11 is also detected at the periphery of the nucleolus. (b) Immuno-EM of control section with omitted primary antibody. Incubation did not reveal any specific labeling within the nucleolus and confirmed the specificity of the immunolabeling. Scale bar = 0.25 µm.

Supplementary Figure 3

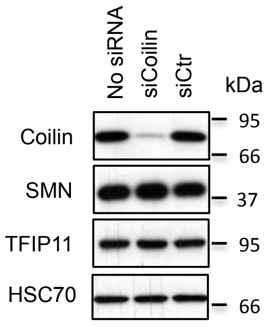

Efficiency of coilin knockdown. HeLa cells were mock-transfected (No siRNA) or transfected with coilin siRNA (siCoilin) or control siRNA (siCtr). Protein extracts were analyzed by western blotting with antibodies against coilin, SMN, TFIP11 and HSC70 (loading control).

Supplementary Figure 4

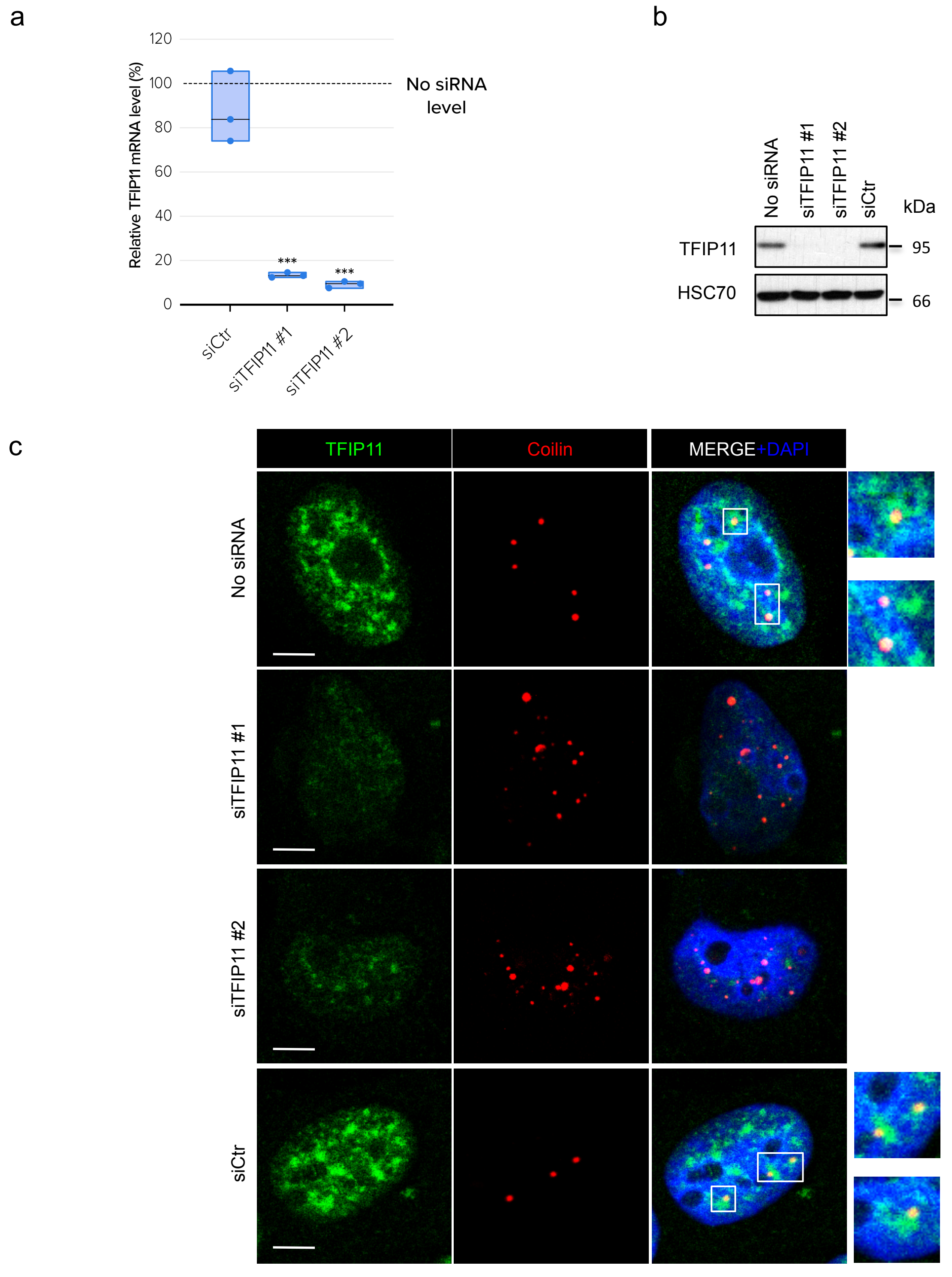

TFIP11 knockdown is efficient and leads to relocalization of coilin. (a) Expression level of TFIP11 mRNA measured by RT-qPCR in HeLa cells mock transfected (No siRNA) or transfected with one of two different siRNAs against TFIP11 (siTFIP11 #1 and siTFIP11 #2) or control siRNA (siCtr). Overall p=4.31e-08 by one-way ANOVA, p=1.1e-06 for siTFIP11#1 and p=2.0e-07 for siTFIP11#2 by Tukey post-hoc test, n=3. Only p-values < 0.05 (relative to siCtr) are displayed. Box limits=min to max; line=median. (b) Protein extracts from HeLa cells transfected as in A were analyzed by western blotting with antibodies against TFIP11 and HSC70 (loading control). (c) TFIP11 and coilin co-staining upon TFIP11 knockdown. HeLa cells transfected as in A were stained with antibody against TFIP11 (in green) and coilin (in red). Individual channels, merged channels with nuclear staining (in blue) and magnification of boxed regions are shown. Scale bar = 5 µm. Source data are provided as a Source Data file.

Supplementary Figure 5

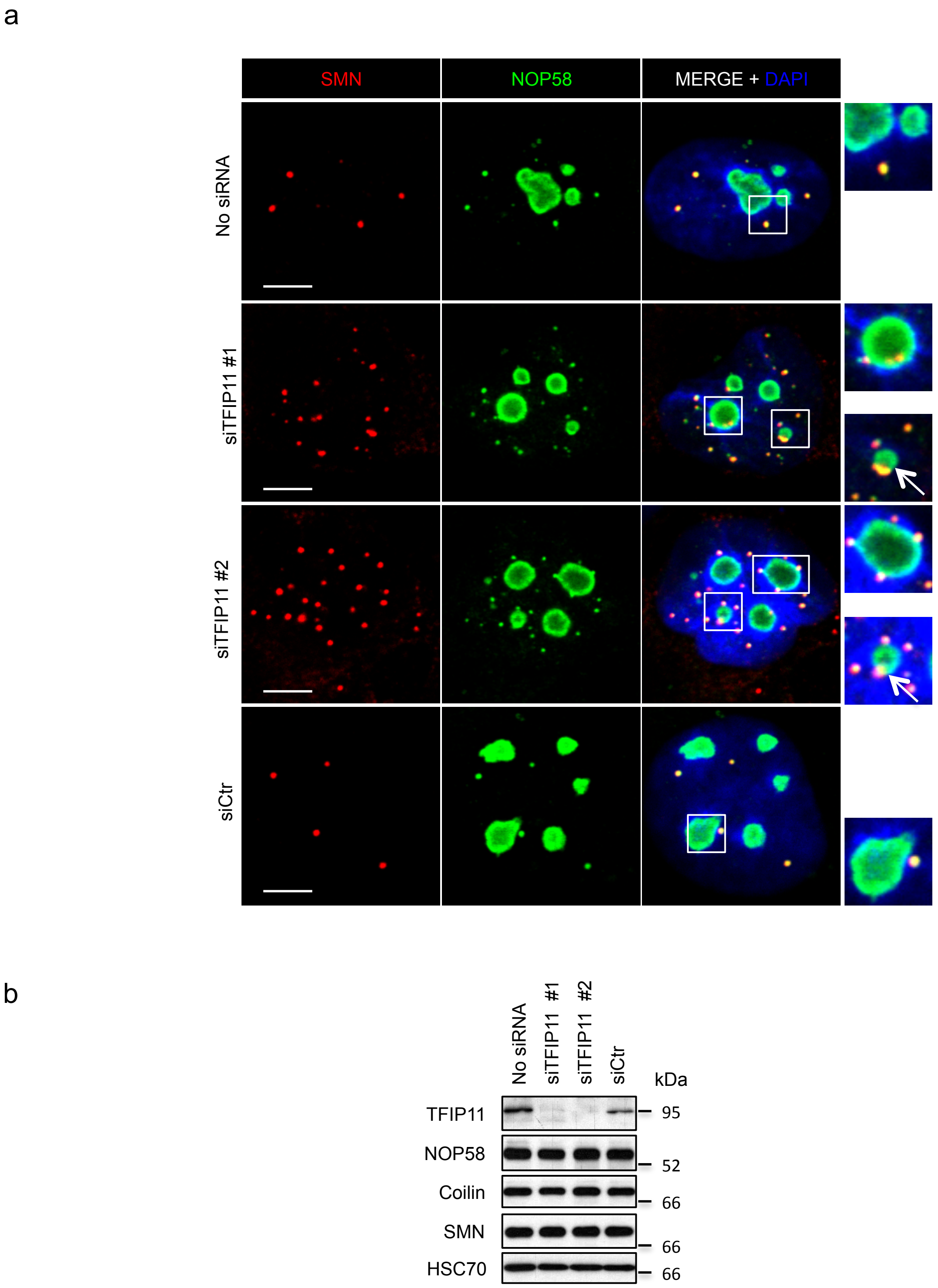

SMN and Nop58 co-staining upon TFIP11 knockdown. (a) HeLa cells were mock-transfected (No siRNA) or transfected with one of two different TFIP11 siRNAs (siTFIP11 #1 and siTFIP11 #2) or control siRNA (siCtr). Cells were co-stained with anti-SMN (in red) and anti-Nop58 (in green) antibodies. Individual channels and merged channel with DAPI counterstaining of the nucleus (in blue) and magnification of boxed regions are shown. Scale bar = 5 µm. (b) TFIP11 knockdown does not affect the expression level of Nop58, SMN and coilin. HeLa cells were transfected as in a. Protein extracts were analyzed by western blotting with antibodies against TFIP11, Nop58, SMN, coilin and HSC70 (loading control).

Supplementary Figure 6

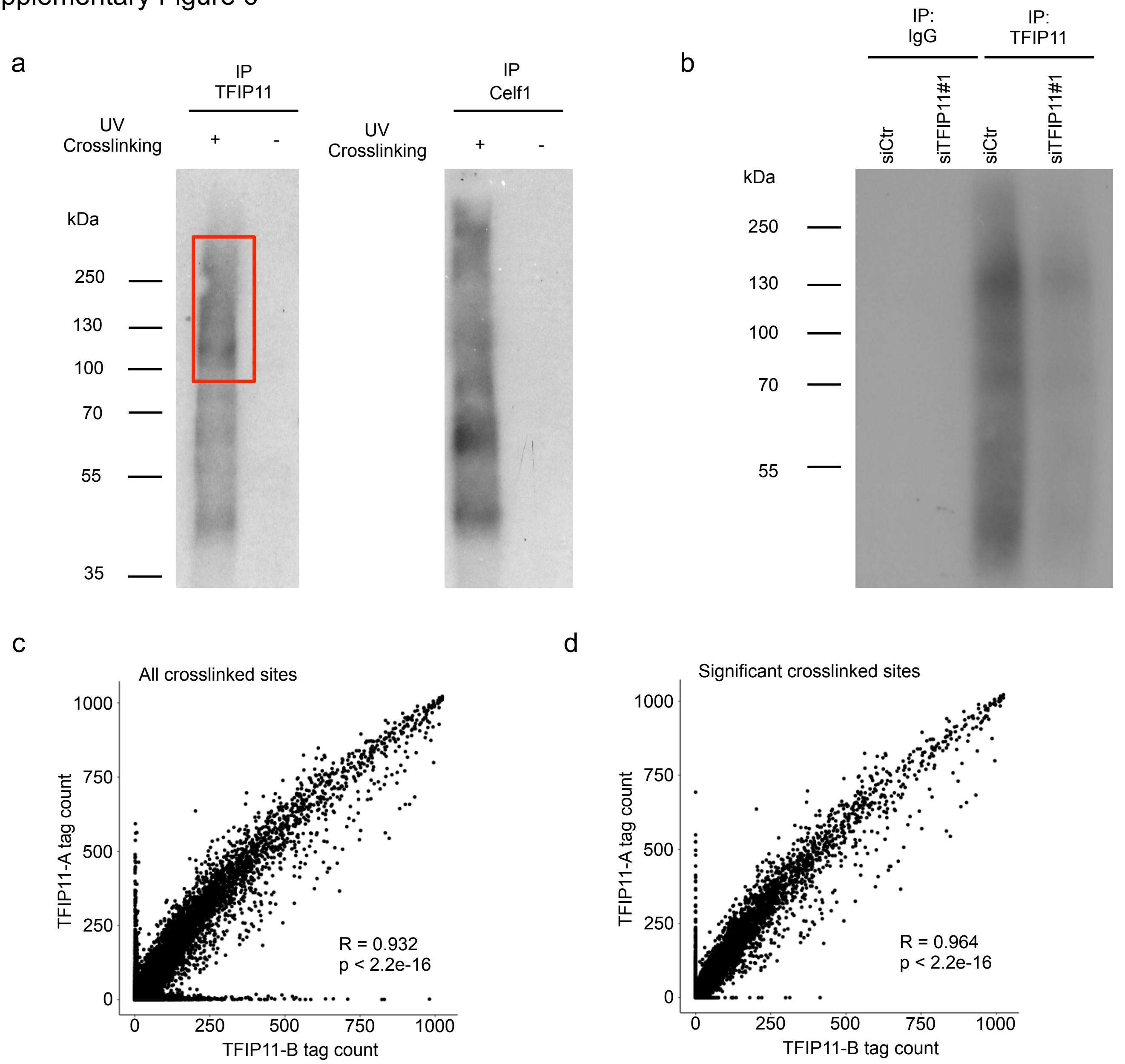

e

Library	Barcode	Input reads	Ave. length	Uniquely mapped	% unique	xlink sites	Significant xlink sites
TFIP11-A	NNNGGTTNN	63,167,668	56	28,456,389	45.05%	2,520,067	403,913
TFIP11-B	NNNTTGTNN	79,353,718	54	33,834,571	42.64%	1,467,429	226,385

Validation of iCLIP-seq experiment. (a) Endogenous TFIP11 was immunoprecipitated (IP) from protein extracts of HeLa cells that had been UV crosslinked (+) or not UV crosslinked (-). The TFIP11-RNA complex was separated using denaturing gel electrophoresis and detected by autoradiography. Red box represents region used to isolate RNA for iCLIP seq analysis. The Celf1-RNA complex was used a positive control. (b) Autoradiography analyses of either TFIP11-RNA or IgG-RNA complexes, using denaturing gel electrophoresis. Immunoprecipitation (IP) of endogenous TFIP11 from protein extracts of HeLa cells transfected with TFIP11 siRNA (siTFIP11 #1) or control siRNA (siCtr). IgG was used as negative IP control. (c) Scatterplot of iCLIP tag depth at all crosslinked sites by nucleotide position in two iCLIP libraries. Pearson correlation and associated p-value are displayed. (d) Scatterplot of iCLIP tag depth at statistically significant crosslinked sites by nucleotide position in two iCLIP libraries. Pearson correlation and associated p-value are displayed. (e) Sequencing and mapping information for two TFIP11 iCLIP libraries (TFIP11-A & TFIP11-B). Source data are provided as a Source Data file.

Supplementary Figure 7

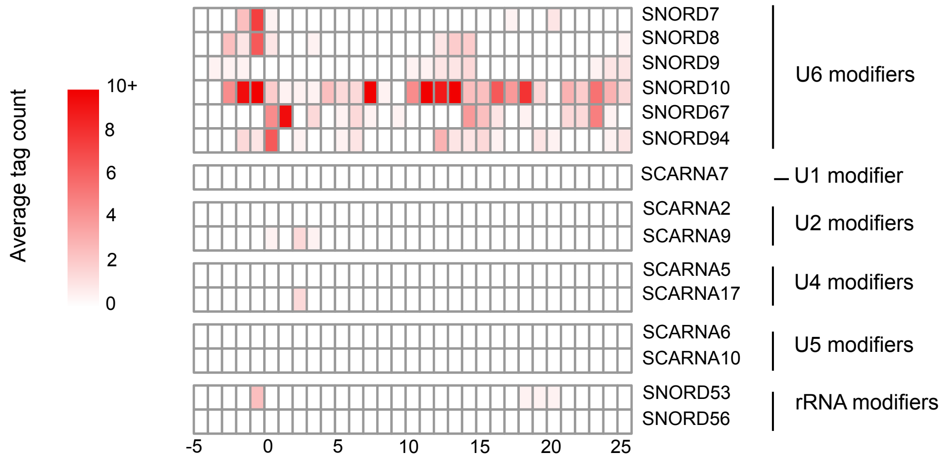

The U6-methylating snoRNAs have more TFIP11 binding at/near their 5' ends than the other U snRNA- or rRNA-methylating snoRNAs. Heatmap of iCLIP tag count at TFIP11 binding sites near 5’ ends of snRNA-modifying and rRNA-modifying sno/scaRNAs. Tag count is averaged over two libraries. Numbers on x-axis indicate nucleotide position relative to 5’ end. Source data are provided as a Source Data file.

Supplementary Figure 8

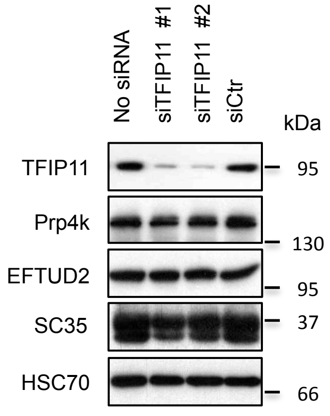

TFIP11 knockdown does not affect the expression level of Prp4K, EFTUD2 or SC35. Protein extracts from HeLa cells mock-transfected (No siRNA) or transfected with one of two different TFIP11 siRNAs (siTFIP11 #1 and siTFIP11 #2) or control siRNA (siCtr) were analyzed by western blotting with antibodies against TFIP11, Prp4K, EFTUD2, SC35 and HSC70 (loading control).

Supplementary Figure 9

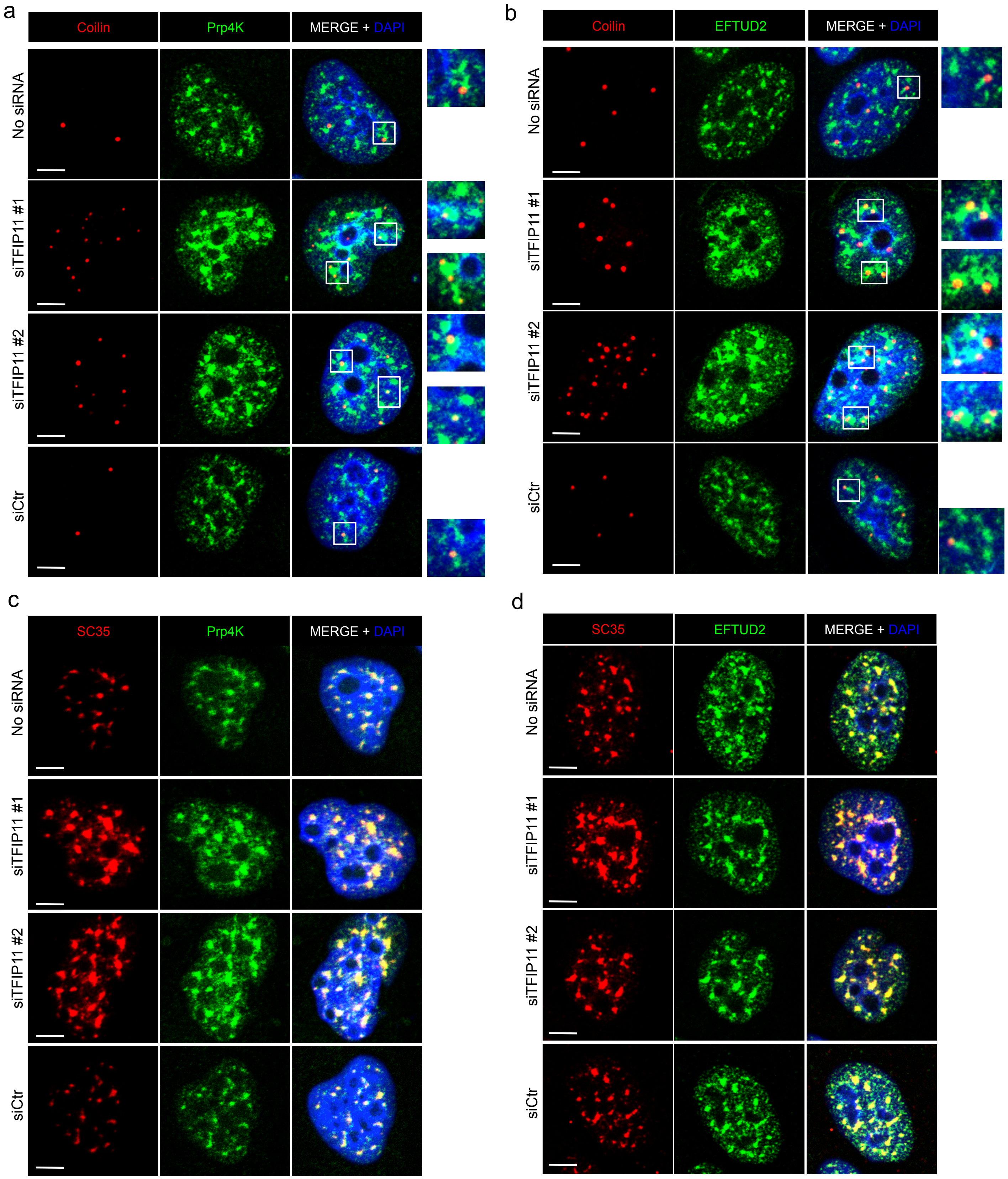

Prp4K or EFTUD2 co-staining with coilin and SC35 upon TFIP11 knockdown. (a-b) HeLa cells were mock-transfected (No siRNA) or transfected for 48 h with one of two different TFIP11 siRNAs (siTFIP11 #1 and siTFIP11 #2) or control siRNA (siCtr). Co-staining with anti-coilin antibody (in red) and antibodies against Prp4K (a) or EFTUD2 (b) (in green) was then performed. Individual channels and merged channels with nuclear staining (in blue) are shown. Scale bar = 5 µm. (c-d) HeLa cells were transfected as in a. Co-staining with anti-SC35 antibody (in red) and antibodies against Prp4K (c) or EFTUD2 (d) (in green) was then performed. Individual channels and merged channels with DAPI counterstaining of the nucleus (in blue) are shown. Scale bar = 5 µm.

Supplementary Figure 10

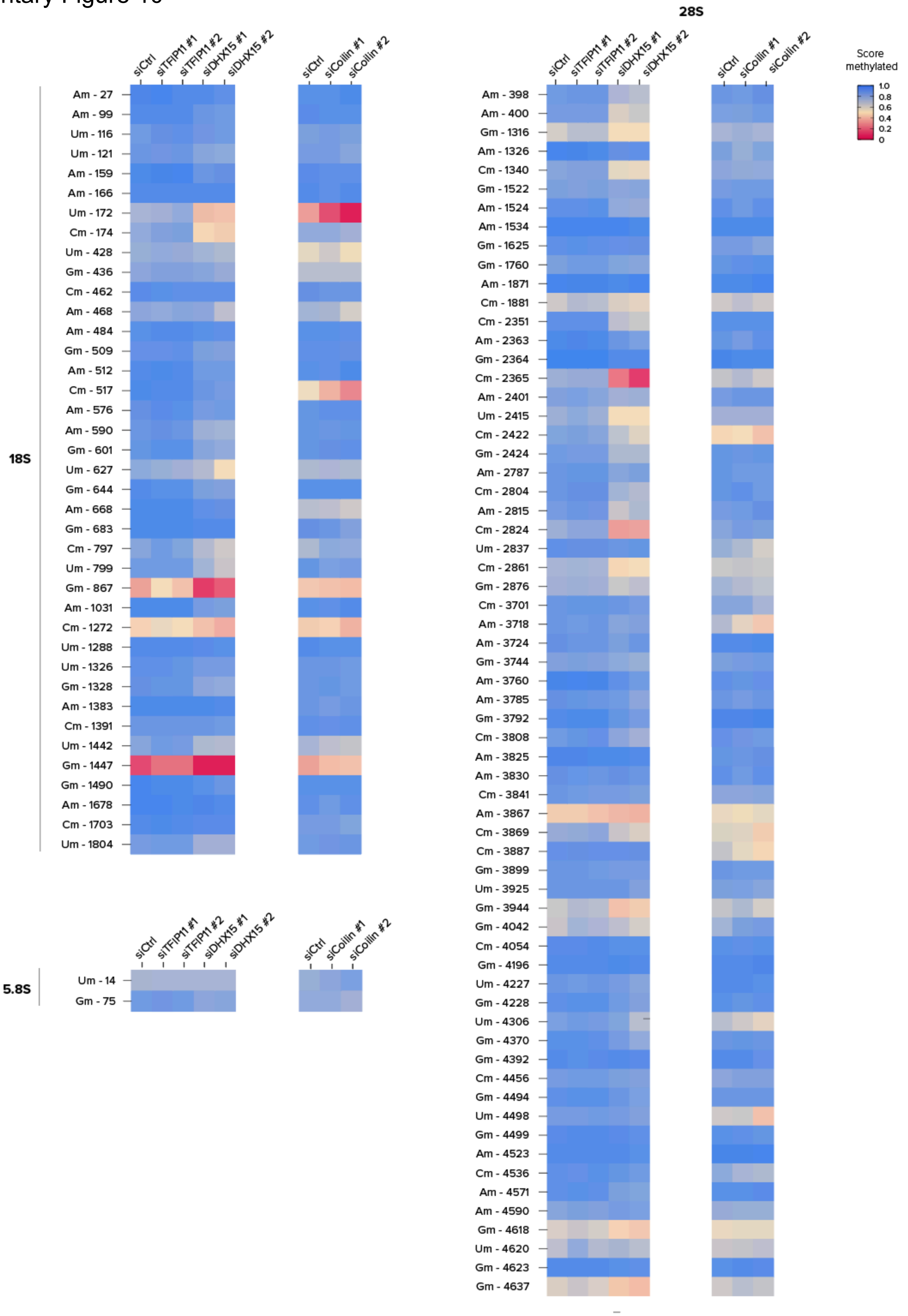

TFIP11 knockdown does not affect the 2'-O-methylation of rRNAs, while knockdown of DHX15 or coilin does. Comparison of 2'-O-methylation of rRNAs between HeLa cells mock transfected (No siRNA) or transfected with control siRNA (siCtr), one of two siRNAs directed against TFIP11 (siTFIP11 #1 and siTFIP11 #2), one of two siRNAs directed against DHX15 (siDHX15 #1 and siDHX15 #2) or one of two siRNAs directed against coilin (siCoilin #1 and siCoilin #2). The top heatmap represents the proportion of methylation for each position indicated on the left on 18S rRNA. The middle heatmap represents the proportion of methylation for each position indicated on the left on 5.8S rRNA and the bottom heatmap on 28S rRNA. Source data are provided as a Source Data file.

Supplementary Figure 11

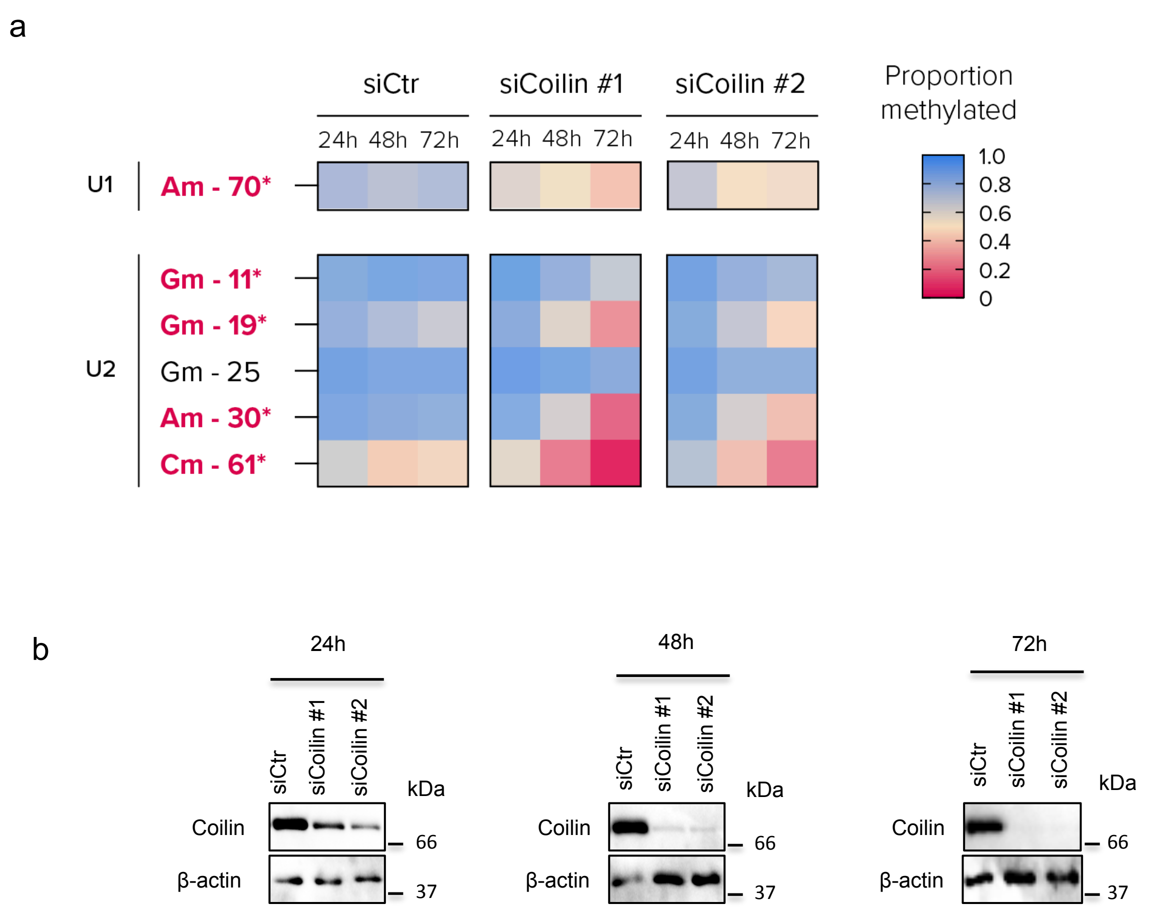

Coilin knockdown decreases the 2'-O-methylation level of U1 and U2 snRNAs in a time-dependent manner. (a) Comparison of 2'-O-methylation on U1 and U2 snRNAs between HeLa cells transfected with siRNA control (siCtr) and HeLa cells transfected for 24, 48 or 72 h with one of two siRNAs directed against coilin (siCoilin #1 and siCoilin #2). Hypomodifications are depicted in red with asterisks. (b) Western blotting showing coilin inhibition and β-actin (loading control) at each time point is shown. Source data are provided as a Source Data file.

Supplementary Figure 12

a

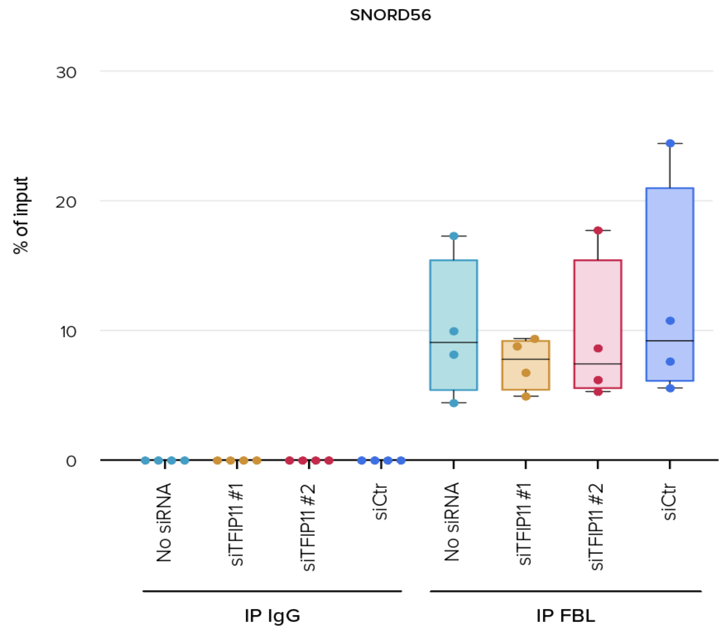

b

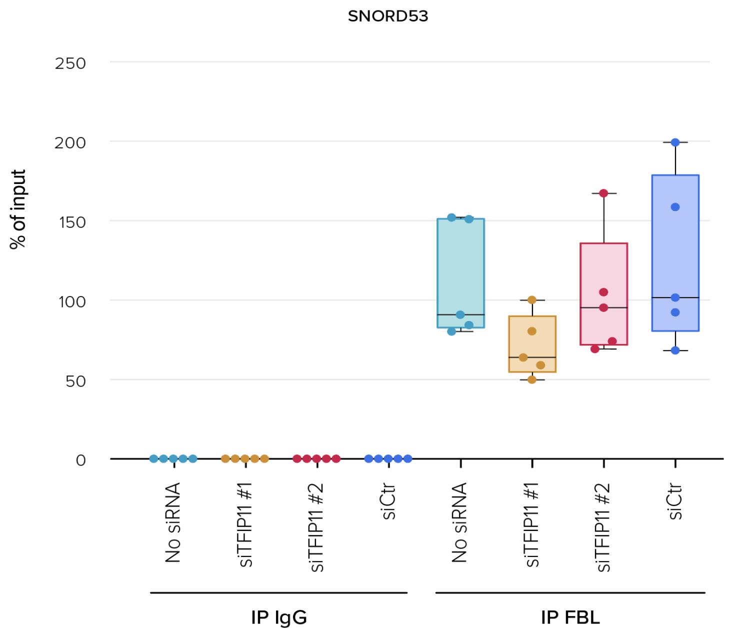

TFIP11 knockdown does not affect the loading of SNORD56 and SNORD53 onto the fibrillarin (FBL) complex. RT-qPCR of SNORD56 (a) and SNORD53 (b) after immunoprecipotation (IP) with an antibody against FBL or control IgG in HeLa cells mock transfected (No siRNA) or transfected with control siRNA (siCtr) or one of two siRNAs against TFIP11 (siTFIP11 #1 and siTFIP11 #2). SNORD56: overall $p = 0.518$ by one-way ANOVA. SNORD53: overall $p = 0.215$ by one-way ANOVA. Only p-values < 0.05 (relative to siCtr) are displayed. Source data are provided as a Source Data file.

Supplementary Figure 13

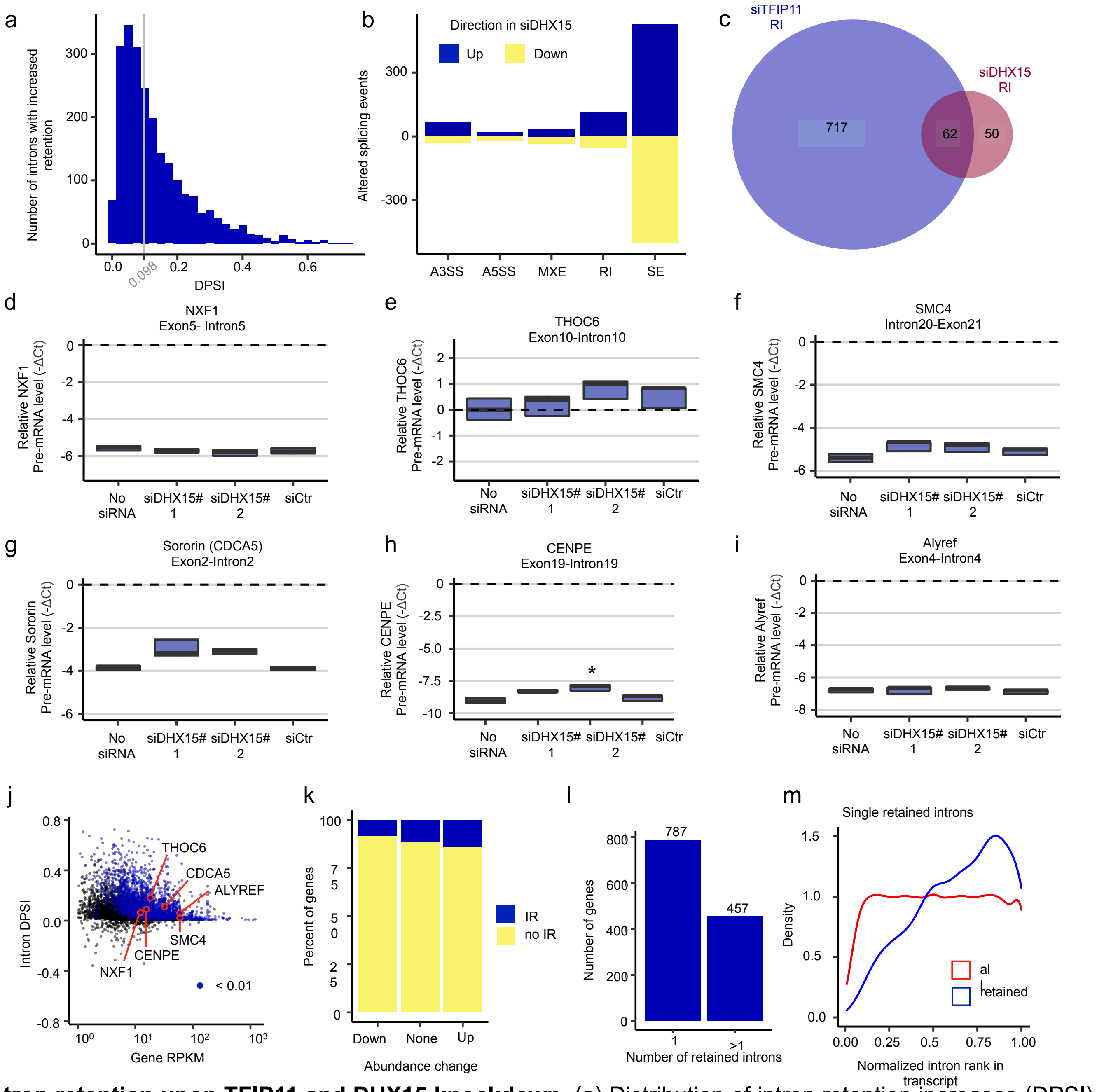

Intron retention upon TFIP11 and DHX15 knockdown. (a) Distribution of intron retention increases (DPSI) for introns showing increased retention detected by IRFinder in cells transfected with siTFIP11 #1 compared to cells transfected with siCtr. Median DPSI = 0.098. (b) Changes in splicing events identified by rMATS in cells transfected with siDHX15 #1 compared to cells transfected with siCtr, including alternative 5' splice site (A5SS), alternative 3' splice site (A3SS), mutually exclusive exon (MXE), retained intron (RI), and skipped exon (SE) events. (c) Venn diagram of introns with increased retention detected by rMATS in cells transfected with siTFIP11 #1 compared to cells transfected with siCtr (blue) and in cells transfected with siDHX15 #1 compared to cells transfected with siCtr (red) (d-i) Evaluation of intron retention by RT-qPCR for selected mRNAs in HeLa cells mock-transfected (No siRNA) or transfected with one of two different siRNAs against DHX15 (siDHX15 #1 and siDHX15 #2) or control siRNA (siCtr). P-values were calculated by ANOVA with Tukey post-hoc test. Only p-values < 0.05 (relative to siCtr) are displayed. Box limits=min to max; line=median. (j) Extent of intron retention change by gene expression level. Each point represents one intron; blue points indicate significant change in intron retention level (Audic-Claverie test statistic < 0.01). DPSI = Difference in Percent Spliced-In. RPKM = Reads Per Kilobase of transcript per Million mapped reads in siCtr-treated cells. (k) Percent of genes showing increased IR, according to gene expression change upon TFIP11 knockdown. (l) Number of introns with increased retention for each gene showing increased IR in HeLa cells depleted for TFIP11. (m) Representation of intron position along transcripts normalized by the total count of introns present in each transcript (i/N, where i is the ith intron and N is the number of introns in the APPRIS primary transcript for that gene). Blue = retained introns in genes showing increased retention of a single intron (n = 787). Red = all introns in genes showing increased retention of a single intron (n = 787 genes, 11,077 introns). Source data are provided as a Source Data file.

Supplementary Figure 14

a

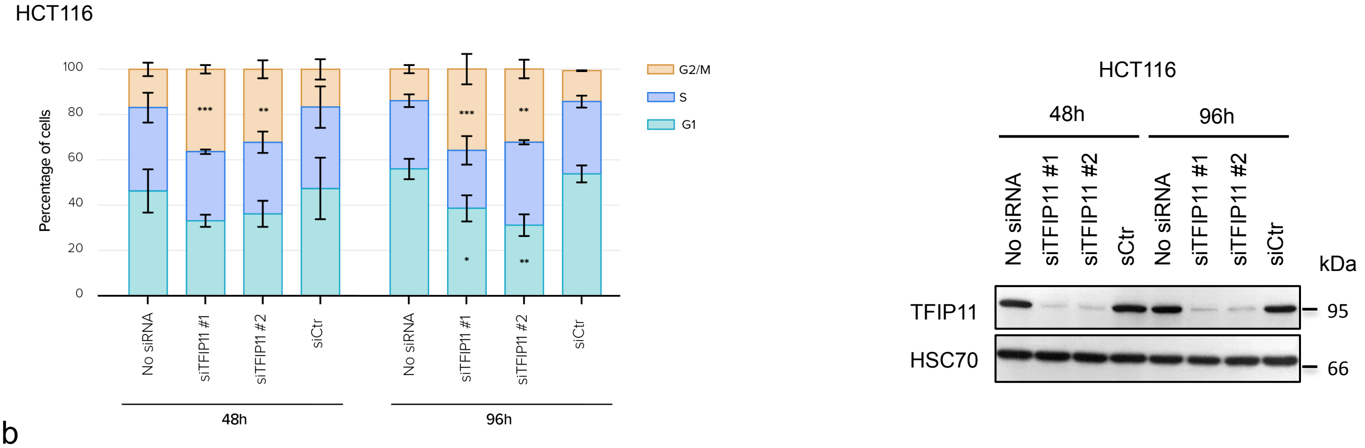

b

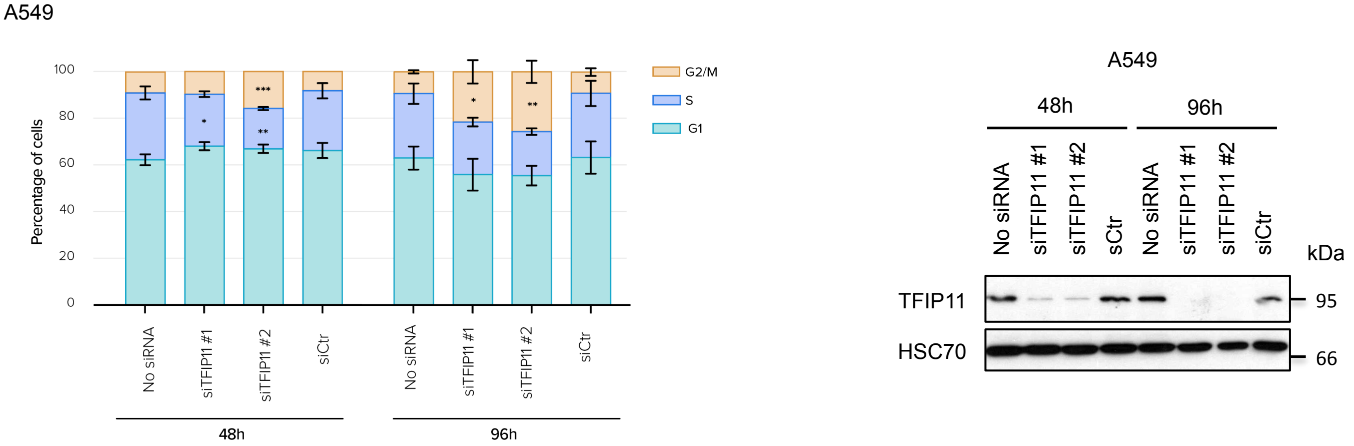

c

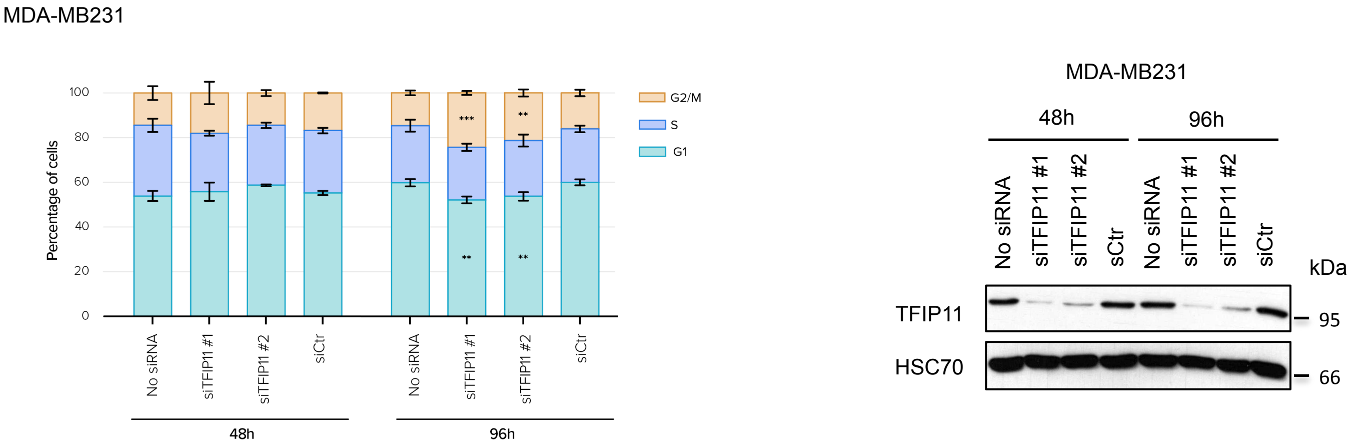

TFIP11 knockdown induces G2/M arrest in several cancer cell lines. Cell cycle analysis of HCT116 colon cancer cells (a), A549 lung cancer cells (b) and MDA-MB231 breast cancer cells (c) mock-transfected (No siRNA) or transfected with one of two different siRNAs against TFIP11 (siTFIP11 #1 and siTFIP11 #2) or control siRNA (siCtr). Flow cytometry analysis (FACS) was performed 48 h and 96 h post-transfection. P-values were calculated by ANOVA with Tukey post-hoc test. Only p-values < 0.05 (relative to siCtr) are displayed. A representative western blotting showing TFIP11 inhibition in each cell type is shown. Source data are provided as a Source Data file.

Supplementary Figure 15

a

HeLa

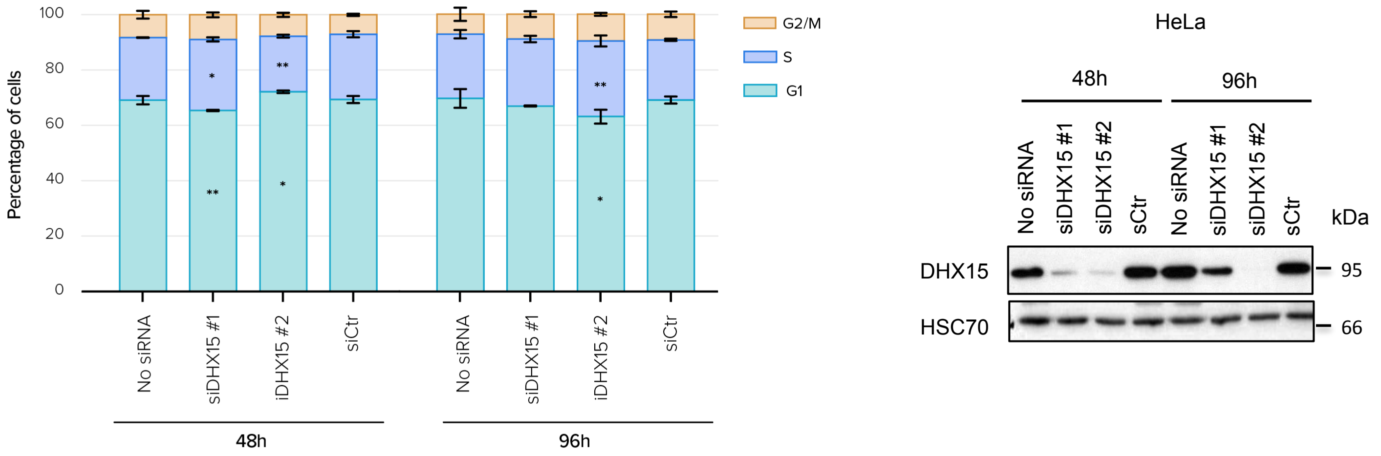

b

A549

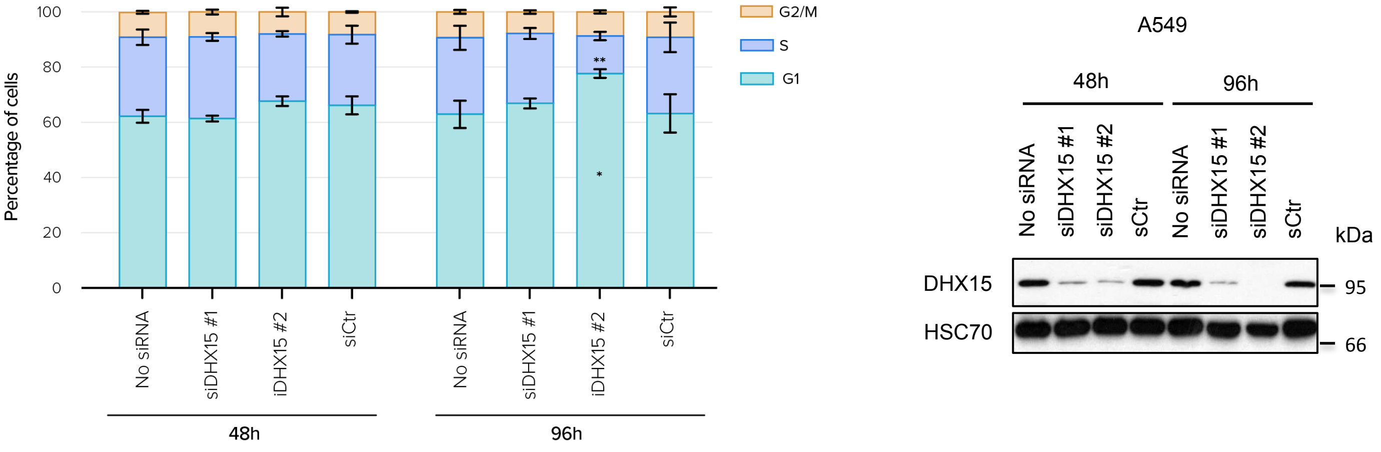

DHX15 knockdown has little impact on cell cycle progression of cancer cell lines. Cell cycle analysis of (a) HeLa cervical cancer cells and (b) A549 lung cancer cells mock-transfected (No siRNA) or transfected with one of two different siRNAs against DHX15 (siDHX15 #1 and siDHX15 #2) or control siRNA (siCtr). Flow cytometry analysis (FACS) was performed 48 h and 96 h post-transfection. P-values were calculated by ANOVA with Tukey post-hoc test. Only p-values < 0.05 (relative to siCtr) are displayed. A representative western blotting showing DHX15 inhibition in each cell type is shown. Source data are provided as a Source Data file.

Supplementary Figure 16

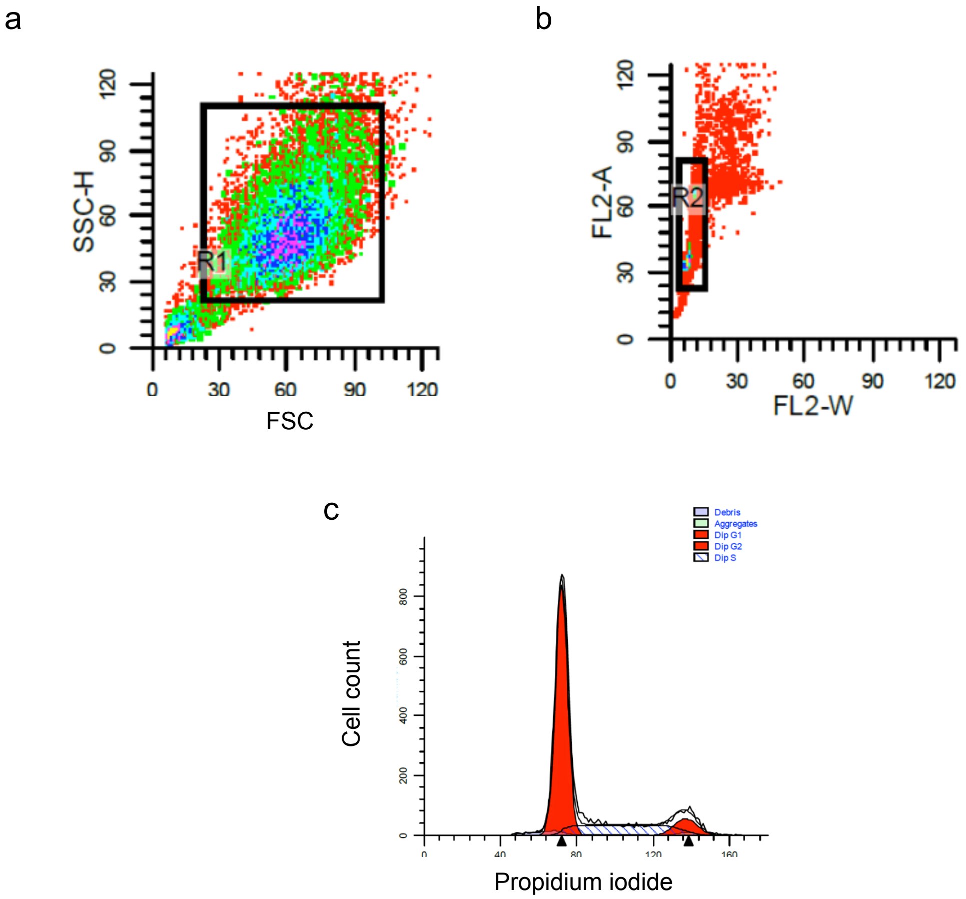

Flow cytometry gating strategy used to identify cell cycle phases. (a) Total cells are gated according to their intrinsic size (FSC) and granularity (SSC) properties on a log scale. The gate excludes cellular debris from living cells. (b) Living cells were then gated based on the area (FL2-A) and the width (FL2-W) of the signal for DNA staining to exclude doublets. (c) The DNA histogram plot obtained after gating on living cells, allowing determination of the percentage of cells in G0-G1 (left red gate), S and G2-M (right red gate) phases using ModFit software.

Supplementary Table 1: siRNA sequences

siRNA	Sense Sequence 5' → 3'	Anti-sense Sequence 3' → 5'
DHX15 #1	GGUUAGACAUCAGUCCUUU	AAAGGACUGAUGUCUAACC
DHX15 #2	GGUUAUAGUUAUGAGCGCUACUCUA	UAGAGUAGCGCUCAUAACUAUAACC
Gl3 (siCtr)	CUUACGCUGAGUACUUCGAUU	AAUCGAAGUACUCAGCGUAAGUU
TFIP11 #1	CCUGUUAAGCAGGACGACU	AGUCGUCCUGCUUAACAGG
TFIP11 #2	GGAUUAGCAAGAAGCUCAC	GUGGCUUCUUGCUAAUCC
Coilin #1	UUUACCACGUCAUUUAAUUGC	GAGAGAACCUGGGAAAUUU
Coilin #2	GAGAGAACCUGGGAAAUUU	AAAUUUCCCAGGUUCUCUC

Supplementary Table 2: Antibodies used in immunofluorescence experiments

Antibody	Company	Catalogue number	RRID	Working dilution
α-tubulin	Cell Signaling	#2125	AB_2619646	1:500
Y-tubulin	Santa Cruz	#sc-51715	AB_630410	1:500
DHX15	Novus Biological	#NBP2-13919	AB_2884951	1:300
SC35	Abcam	#ab11826	AB_298608	1:500
TFIP11	Bethyl Lab	#A302-549A	AB_1999068	1:250
TFIP11	Proteintech	#66114-Ig	AB_2881513	1:250
coilin	Abcam	#ab87913	AB_10860831	1:400
SMN	BD Biosciences	#610646	AB_397973	1:250
Fibrillarin	ABclonal	#A1136	AB_2758523	1:250
NOP58	ABclonal	#A4749	AB_2765846	1:250
EFTUD2	Bethyl Lab	#A300-957A-T	AB_2779577	1:250
PRPF4B (Prp4K)	Bethyl Lab	#A301-665A-T	AB_2780042	1:250
Secondary antibodies conjugated with Alexa dyes	Thermo Fisher Scientific	Goat anti Rabbit IgG Alexa Fluor 488 conjugate #A-11034<br>Donkey anti-rabbit IgG, Alexa Fluor 488 conjugate #A-21206<br>Goat anti-Mouse	AB_2576217<br>AB_2535792	All used 1:2000

		IgG, Alexa Fluor 488 conjugate #A-11001<br>Donkey anti-Mouse IgG, Alexa Fluor 546 conjugate #A-10036<br>Goat anti-rabbit IgG, Alexa Fluor 546 conjugate #A-11035	AB_2534069<br>AB_2534012<br>AB_143051	

Supplementary Table 3: U snRNA probes used in northern blotting experiments

U1	CATCCGGAGTGCAATGGATAAGCCTCGCCCTGGGAAAACCACCTTCGTG ATCATGGTATCTCCCCTGCCAGGTAAGTAT
U2	ATTGTCCTCGGATAGAGGACGTATCAGATATTAAACTGATAAGAACAGATA CTACACTTGATCTTAGCCAAAAGGCCGAG
U4	CGGGGTATTGGGAAAAGTTTTCAATTAGCAATAATCGCGCCTCGGATAAA CCTCATTGGCTACGATACTGCCACTGCGCAAAGCT
U5	TTGGGTTAAGACTCAGAGTTGTTCCTCTCCACGGAAATCTTTAGTAAAAGG CGAAAGATTTATGCGATCTG
U6	GGAACGCTTCACGAATTTGCGTGTCATCCTTGCGCAGGGGCCATGCTAAT CTTCTCTGTATCGTTCCA

Supplementary Table 4: Antibodies used in western blotting experiments

Antibody	Company	Catalogue number	RRID	Working dilution
β-actin	Santa Cruz	#sc-69879	AB_2714189	1:5000
Aurora A	Anticorps-online	#ABIN4956113	AB_2884953	1:1000
P-Aurora A	Cell Signaling	#2914	AB_2061631	1:1000
CENPE	Abcam	#ab5093	AB_304747	1:2000
Cyclin B1	Santa Cruz	#sc-594	AB_2890159	1:1000
Histone H3	Millipore	#06-755	AB_2118461	1:3000
P-H3 (Ser10)	Cell Signaling	#3377	AB_1549592	1:2000
DHX15	Novus Biological	#NBP2-13919	AB_2884951	1:2000
NXF1	Cell Signaling	#12735	AB_2798011	1:1000
HSC70	Santa Cruz	#sc-7298	AB_627761	1:5000
Sororin	Kind gift from Jan-Michael Peters'Lab			1:1000
SC35	Abcam	#ab11826	AB_298608	1:3000
TFIP11	Bethyl Lab	#A302-549A	AB_1999068	1:2000
TFIP11	Proteintech	#66114-Ig	AB_2881513	1:2000
coilin	Proteintech	#10967-1-AP	AB_2276345	1:1000
coilin	Abcam	#ab87913	AB_10860831	1:1000
SMN	BD	#610646	AB_397973	1:2000

	Biosciences			
SMN	Proteintech	#11708-1-AP	AB_2255114	1:1000
Fibrillarin	Bethyl Lab	#A303-891A	AB_2620241	1:2000
NOP58	ABclonal	#A4749	AB_2765846	1:1000
EFTUD2	Bethyl Lab	#A300-957A-T	AB_2779577	1:2000
PRPF4B (Prp4K)	Cell Signaling	#8577	AB_10897513	1:1000
FLAG	Sigma	#F7425	AB_439687	1:5000
THOC6	Proteintech	#14168	AB_2240482	1:2000
Prp3	Bethyl Lab	#A302-073A	AB_1604202	1:2000
Prp6	Bethyl Lab	#A302-774A-T	AB_10630102	1:2000
Prp8	Bethyl Lab	#A303-922A-T	AB_2781571	1:2000
PRPF31	Bethyl Lab	#A303-919A	AB_2620268	1:1000
SART3	Novus Biological	#NB120-10435	AB_2110146	1:1000
SMC4	Cell Signaling	#5547	AB_10698892	1:1000
SNRNP200	Bethyl Lab	#A304-635A	AB_2620830	1:2000
Secondary antibodies conjugated with horseradish peroxidase (HRP)	Anti-rabbit HRP Antibody<br><br>Anti-Mouse HRP Antibody	Cell Signaling #7074<br><br>Dako #P0260	AB_2099233<br><br>AB_2636929	All used 1:5000 to 1:10,000

Supplementary Table 5: Antibodies used in co-immunoprecipitation and RIP experiments

Antibody	Company	Catalogue number	RRID	Working dilution
SNRPB (Sm)	Invitrogen	#MA5-13449	AB_10944191	2 µg per sample
TFIP11	Proteintech	#66114-Ig	AB_2881513	2 µg per sample
coilin	Proteintech	#10967-1-AP	AB_2276345	2 µg per sample
FLAG	Sigma	#F1804	AB_262044	2 µg per sample
Fibrillarin	Bethyl Lab	#A303-891A	AB_2620241	RIP: 3-4 µg per sample<br>IP: 2 µg per sample

Supplementary Table 6: Sequences of primers used in qPCR experiments

mRNA	Reverse sequence 5' → 3'	Forward Sequence 5' → 3'
β-actin	AGAGGCGTACAGGGATAGCA	AGAAAATCTGGCACCACACC
Alyref	ATTGGACACCAGCAGTTTCCC	GCACGATCTTTTCGACAGTGG
Alyref intron 4	GAAGGACCCTAAGAGCGACG	TCACGTCACAGATTGACGCA
CENPE	AAGCACCCAAACTCGAATCA	ACCCTTCATATGGACTTTGAGCA
CENPE intron 19	TGAGAAAAACATTGCAAATGCACAA	GCCCTTGATGCCAAGAGAGA
DHX15	CCAGGATCAATCACAAACACCACAC	TCTACACTTCCACCTCAGCAGCA
NXF1	CCCCGGCCTTTCTTCTTTCT	GAGCGCTTGGGAGTTAGGTT
NXF1 intron 5	AAGCCTAAGCCGCAAGAACA	AGAATACACGGGCCCAGTTC
SMC4	GTTGGCAGGCATTTCCAGTG	ACCTGGAGGGTCAGGTAACTT
SMC4 intron 20	TCCTCTGGGCTTAGAACCGA	GGAGGGTTTGGGGAGGATTG
Sororin	TCGAAGCCAAAGCAGGAC	AAGTCAGGCGTTCCTACAGC
Sororin intron	AGAGGGATGAACGTGAGCTC	AGGGCCCCAUCUCCUACUAA

2		
Snord7	AGCAATGGCAATACCAGGTC	TGCGATGATGAGTGAAGTAGAG
Snord 67	TGTCACCAGGGTGATACTGA	GTGAGAGTGATGAGTTGCACA
Snord 94	GAGGCAACAGTCTCATGTAAGT	AGTCATGGGAGCTGAATGTATG
Snord 56	CACTCAGACCCAGAGTCTCAAC	CCACAATGATGGCAATATTTTTC GT
Snord 53	GCTCAGACAGCCAAGAGAAAG	TGATGACATCCATATGGTTTCGC TG
TFIP1 1	ACCATCGGGTCACAGTTCC	GCAGATGCCTTTCACAGGTT
THOC 6	AGAAGGACAGGGAGAAGGCT	CTGACAGCTGCAGGCAACA
THOC 6 intron 10	CTGCCATCCCATCCCACAGTC	GCTGGATTGGATGTTTGGCAAC
U6	AAAAATATGGAACGCTTCACGAA	CGCCAGCACATATACTAAAATTG G
