# Supplementary material for: DHX15-independent roles for TFIP11 in U6 snRNA modification, U4/U6.U5 tri-snRNP assembly and pre-mRNA splicing fidelity
Source: Nat Commun. 2021 Nov 17;12:6648. doi: 10.1038/s41467-021-26932-2 (PMC8599867; doi:10.1038/s41467-021-26932-2)
Supplement: Supplementary file 2 — Description of Additional Supplementary Files [file 41467_2021_26932_MOESM2_ESM.pdf]

Description of Additional Supplementary Files

File Name: Supplementary Data 1
Description: snRNA, snoRNA and scaRNA expression changes upon TFIP11 depletion. Differential expression information for snRNA, snoRNA and scaRNA transcripts in cells transfected with one siRNA targeting TFIP11 (TFIP11 #1) compared with cells transfected with control siRNA (siCtr)

File Name: Supplementary Data 2
Description: GO terms and genes with IR upon TFIP11 depletion. GO terms enriched in genes with increased intron retention in cells transfected with one siRNA targeting TFIP11 (TFIP11 #1) compared with cells transfected with control siRNA (siCtr)
