# Supplementary material for: DHX15-independent roles for TFIP11 in U6 snRNA modification, U4/U6.U5 tri-snRNP assembly and pre-mRNA splicing fidelity
Source: Nat Commun. 2021 Nov 17;12:6648. doi: 10.1038/s41467-021-26932-2 (PMC8599867; doi:10.1038/s41467-021-26932-2)
Supplement: Supplementary file 5 — Reporting Summary [file 41467_2021_26932_MOESM5_ESM.pdf]

nature research

Corresponding author(s): Pr. Denis Mottet

Last updated by author(s): 09/30/2021

Reporting Summary

Nature Research wishes to improve the reproducibility of the work that we publish. This form provides structure for consistency and transparency in reporting. For further information on Nature Research policies, see our Editorial Policies and the Editorial Policy Checklist.

Please do not complete any field with "not applicable" or n/a. Refer to the help text for what text to use if an item is not relevant to your study.
For final submission: please carefully check your responses for accuracy; you will not be able to make changes later.

Statistics

For all statistical analyses, confirm that the following items are present in the figure legend, table legend, main text, or Methods section.

n/a	Confirmed	
☐	☒	The exact sample size (*n*) for each experimental group/condition, given as a discrete number and unit of measurement
☐	☒	A statement on whether measurements were taken from distinct samples or whether the same sample was measured repeatedly
☐	☒	The statistical test(s) used AND whether they are one- or two-sided<br>*Only common tests should be described solely by name; describe more complex techniques in the Methods section.*
☒	☐	A description of all covariates tested
☒	☐	A description of any assumptions or corrections, such as tests of normality and adjustment for multiple comparisons
☐	☒	A full description of the statistical parameters including central tendency (e.g. means) or other basic estimates (e.g. regression coefficient) AND variation (e.g. standard deviation) or associated estimates of uncertainty (e.g. confidence intervals)
☐	☒	For null hypothesis testing, the test statistic (e.g. *F*, *t*, *r*) with confidence intervals, effect sizes, degrees of freedom and *P* value noted<br>*Give P values as exact values whenever suitable.*
☒	☐	For Bayesian analysis, information on the choice of priors and Markov chain Monte Carlo settings
☒	☐	For hierarchical and complex designs, identification of the appropriate level for tests and full reporting of outcomes
☒	☐	Estimates of effect sizes (e.g. Cohen's *d*, Pearson's *r*), indicating how they were calculated

Our web collection on statistics for biologists contains articles on many of the points above.

Software and code

Policy information about availability of computer code

Data collection

FACS: Modfit.3.2.1; CellQuest 3.2. **Microscopy:** Leica LAS AF Lite 2.7.4.10100

Data analysis

Microscopy analysis: ImageJ 1.51; Imaris 8.
RNA-Seq: FastQC 0.11.3; bbduk 35.59; STAR 2.5.2b; Picard tools 1.139; featureCounts 1.4.6-p5; edgeR 3.14.0; IRFinder 1.2.3; rMATS 3.3.2; DAVID 6.8; R 3.3.1; R 3.3.1; dplyr 1.0.2; ggplot2 3.3.2; tidyr 1.1.2; biomaRt 2.30.0; ggseqlogo 0.1; BedTools v2.25.0; stringr 1.4.0; Biostrings 2.42.1; APPRIS 2017_10.v25; MaxEntScan 2018-10-22; BPfinder 2016; tximport v1.2.0; DESeq2 1.14.1
iCLIP-Seq: iCount 2.0.0; IGV 2.5.2; dplyr 1.0.2; ggplot2 3.3.2; tidyr 1.1.2; rtracklayer 1.34.2; biomaRt 2.30.0; SuperExactTest. 1.0.7; snoReport 2.0
RiboMeth-Seq: snakemake-minimal 5.24.1; samtools 1.9; bowtie2 2.4; FastQC 0.11; multiqc 1.9; Trimmomatic 0.36; bowtie2; R 3.3.1; RNAmodR.RiboMethSeq 1.6.0; snoRNAdb 2016; EpiTxDb v1.3.3
Northern blot detection /Acquisition: Fuji FLA-700 phosphorimager 1.1; Multi Gauge software 3.1
Intrinsic disorder measurements: MFDp2 2.00; PrDOS 2.0; DISOPRED2 3.2; PONDR 2007
Figures: Microsoft Powerpoint 14.7.2, 14.7.7 and 16.53; Adobe Photoshop.13.0; Adobe Illustrator CC 2021; GraphPad Prism 2.2.0

For manuscripts utilizing custom algorithms or software that are central to the research but not yet described in published literature, software must be made available to editors and reviewers. We strongly encourage code deposition in a community repository (e.g. GitHub). See the Nature Research guidelines for submitting code & software for further information.

Data

Policy information about availability of data

All manuscripts must include a data availability statement. This statement should provide the following information, where applicable:

- Accession codes, unique identifiers, or web links for publicly available datasets
- A list of figures that have associated raw data
- A description of any restrictions on data availability

Sequencing data (iCLIP-Seq, RiboMethSeq and RNA-Seq) generated in this study have been deposited in the NCBI GEO database under accession code GSE156390 [https://www.ncbi.nlm.nih.gov/geo/query/acc.cgi?acc=GSE156390] and are publicly available. Other Source data generated in this study are provided in a source data file.

Field-specific reporting

Please select the one below that is the best fit for your research. If you are not sure, read the appropriate sections before making your selection.

☒ Life sciences ☐ Behavioural & social sciences ☐ Ecological, evolutionary & environmental sciences

Life sciences study design

All studies must disclose on these points even when the disclosure is negative.

Sample size	No sample size calculation was performed. All experiments were performed using sample size based on standard protocols in the field. Unless otherwise noted, n=3 biological replicates were performed.
Data exclusions	No data were excluded.
Replication	All experiments were repeated at least 2 or 3 three times. We have specified the number of biological replicates in the figure legends and/or "Statistics and Reproducibility" section, as appropriate. All attempts at replication were successful
Randomization	For each experiment, the same number of cells were initially seeded from the same flask and well-mixed prior to distribution.<br>We randomly took images of fluorescence, TEM and chromosome spread staining under the microscope.<br>Except for that, no randomization was explicitly performed because the experiments were highly reproducible and independently performed by several researchers.<br>The research staff was not blinded to sample allocation because the results were obtained by objective quantitative methods of molecular and cellular biology. The experiments have been repeated by multiple members of the research team and reliably reproduced. Investigators were only blinded to sample identity when analysing IF images and scoring chromosomal abnormalities in order to reduce bias in quantifying the different profiles.
Blinding	The research staff was not blinded to sample allocation because the results were obtained by objective quantitative methods of molecular and cellular biology. The experiments have been repeated by multiple members of the research team and reliably reproduced. Investigators were only blinded to sample identity when analysing IF images and scoring chromosomal abnormalities in order to reduce bias in quantifying the different profiles.

Behavioural & social sciences study design

All studies must disclose on these points even when the disclosure is negative.

Study description	
Research sample	
Sampling strategy	
Data collection	
Timing	
Data exclusions	
Non-participation	
Randomization	

Ecological, evolutionary & environmental sciences study design

All studies must disclose on these points even when the disclosure is negative.

Study description	
Research sample	
Sampling strategy	
Data collection	
Timing and spatial scale	
Data exclusions	
Reproducibility	
Randomization	
Blinding	

Did the study involve field work? Yes ☐ No ☐

Field work, collection and transport

Field conditions	
Location	
Access & import/export	
Disturbance	

Reporting for specific materials, systems and methods

We require information from authors about some types of materials, experimental systems and methods used in many studies. Here, indicate whether each material, system or method listed is relevant to your study. If you are not sure if a list item applies to your research, read the appropriate section before selecting a response.

Materials & experimental systems

n/a	Involved in the study	
☐	☒	Antibodies
☐	☒	Eukaryotic cell lines
☒	☐	Palaeontology and archaeology
☒	☐	Animals and other organisms
☒	☐	Human research participants
☒	☐	Clinical data
☒	☐	Dual use research of concern

Methods

n/a	Involved in the study	
☒	☐	ChIP-seq
☐	☒	Flow cytometry
☒	☐	MRI-based neuroimaging

Antibodies

Antibodies used

Immunofluorescence (IF)
α-tubulin (Cell Signaling #2125, RRID: AB_2619646); ϒ-tubulin (Santa Cruz #sc-51715, RRID: AB_630410); DHX15 (Novus Biological #NBP2-13919, RRID: AB_2884951); SC35 (Abcam #ab11826, RRID: AB_298608); TFIP11 (Bethyl Lab #A302-549A, RRID: AB_1999068,); TFIP11 (Proteintech #66114-Ig, RRID: AB_2881513,); Coilin (Abcam #ab87913, RRID: AB_10860831,); SMN (BD Biosciences #610646, RRID: AB_397973); Fibrillarin (ABclonal #A1136 RRID, AB_2758523,); NOP58 (ABclonal #A4749, RRID: AB_2765846); EFTUD2 (Bethyl Lab #A300-957A-T, RRID: AB_2779577); PRPF4B (Bethyl Lab #A301-665A-T, RRID: AB_2780042,); SART3 (MyBiosource #611684, RRID: AB_2884952); Goat anti Rabbit IgG Alexa Fluor 488 conjugate (Thermo Fisher Scientific #A-11034, RRID: AB_2576217,); Donkey anti-rabbit IgG, Alexa Fluor 488 conjugate (Thermo Fisher Scientific #A-21206, RRID: AB_2535792); Goat anti-Mouse IgG, Alexa Fluor 488 conjugate (Thermo Fisher Scientific #A-11001, RRID: AB_2534069); Donkey anti-Mouse IgG, Alexa Fluor 546 conjugate (Thermo Fisher Scientific #A-10036, RRID: AB_2534012,); Goat anti-rabbit IgG, Alexa Fluor 546 conjugate (Thermo Fisher Scientific #A-11035, RRID: AB_143051)

Western blotting (WB)
-actin (Santa Cruz #sc-69879, RRID: AB_2714189); Aurora A (Anticorps-online #ABIN4956113, RRID: AB_2884953,); P-Aurora A (Cell Signaling #2914, RRID: AB_2061631); Histone H3 (Millipore #06-755, RRID: AB_2118461); P-H3 (Ser10) (Cell Signaling #3377, RRID: AB_1549592); DHX15 (Novus Biological #NBP2-13919, RRID: AB_2884951); HSC70 (Santa Cruz #sc-7298, RRID: AB_627761); SC35 (Abcam #ab11826, RRID: AB_298608); TFIP11 (Bethyl Lab #A302-549A, RRID: AB_1999068); TFIP11 (Proteintech #66114-Ig, RRID: AB_2881513); Coilin (Proteintech #10967-1-AP, RRID: AB_2276345); Coilin (Abcam #ab87913, RRID: AB_10860831); SMN (BD Biosciences #610646, RRID:AB_397973); SMN (Proteintech #11708-1-AP, RRID: AB_2255114) , Fibrillarin (Bethyl Lab #A303-891A, RRID: AB_2620241); NOP58 (ABclonal #A4749, RRID: AB_2765846); EFTUD2 (Bethyl Lab #A300-957A-T, RRID: AB_2779577); PRPF4B (Prp4K) (Cell Signaling #8577, RRID: AB_10897513,); SART3 (Novus Biological #NB120-10435, RRID: AB_2110146,); FLAG (Sigma #F7425, RRID: AB_439687); PRPF31(Bethyl Lab #A303-919A, RRID: AB_2620268); SNRNP200(Bethyl Lab #A304-635A, RRID: AB_2620830); Prp3 (Bethyl Lab #A302-073A, RRID: AB_1604202); Prp6 (Bethyl Lab #A302-774A, RRID: AB_10631979,); Prp8 (Bethyl Lab #A303-922A, RRID: AB_2620269); SMC4 (Cell Signaling #5547, RRID: AB_10698892); NXF1(Cell Signaling #12735, RRID:AB_2798011), CENPE (Abcam #ab5093, RRID:AB_304747); THOC6 (Proteintech #15316-1-AP, RRID:AB_2240482); Cyclin B1(Santa Cruz #sc-752, RRID:AB_627338); Sororin (kind gift from Jan Michael Peters Lab), Anti-rabbit IgG, HRP-linked Antibody Cell Signaling #7074, RRID: AB_2099233,); Rabbit Anti-Mouse Immunoglobulins/HRP antibody (Dako #P0260RRID: AB_2636929)

Co-Immunoprecipiation (Co-IP)
SNRPB (Sm) (Invitrogen #MA5-13449, RRID: AB_10944191); TFIP11 (Proteintech #66114-Ig, RRID: AB_2881513,); TFIP11 (Bethyl Lab # A302-549A, RRID:AB_1999068); Coilin (Proteintech #10967-1-AP, RRID: AB_2276345); FLAG (Sigma #F1804, RRID: AB_262044); Fibrillarin (Bethyl Lab #A303-891A, RRID: AB_2620241)

RNA-Immunoprecipitation (RIP)
Fibrillarin (Bethyl Lab #A303-891A, RRID: AB_2620241)

iCLIP-Seq
TFIP11 (Bethyl Lab # A302-549A, RRID: AB_1999068)

Validation	All antibodies, except for polyclonal anti-Sororin, are commercially available and have been validated by the manufacturers. Detailed information can be found on the website from the manufacturers using catalog numbers provided in Supplementary Tables 2, 4 and 5. In addition, some antibodies were validated by siRNA knockdown or plasmid overexpression. Anti-Sororin rabbit antibody is a kind gift from Jan-Michael Peters Lab and has been used in western blotting applications in several publications (Nishiyama T et al., Cell 2010, PMID: 21111234 - Nishiyama T et al., PNAS 2013, PMID: 23901111 - van der Lelij P et al. EMBO J 2014, PMID: 25257309 - Ladurner R et al., EMBO J 2016, PMID: 26903600).

Eukaryotic cell lines

Policy information about cell lines

Cell line source(s)	All cell lines are from ATCC: HeLa (ATCC, #CCL-2 – RRID:CVCL_0030), HCT116 (ATCC, #CCL-24 - RRID:CVCL_0291), A549 (ATCC, #CCL-185 - RRID:CVCL_0023), MDA-MB-231 (ATCC, #HTB-26 - RRID:CVCL_0062) and U2OS cells (ATCC, #HTB-96 - RRID:CVCL_0042)
Authentication	Cell lines were not authenticated as they were purchased directly from suppliers. Each cell line was routinely examined by microscope to check shape, growth conditions, proliferation and the presence of any contaminants.
Mycoplasma contamination	All cells were routinely (every two weeks) examined for mycoplasma contamination by MycoAlertTM Mycoplasma Detection Kit (Lonza, #LT07-118
Commonly misidentified lines (See ICLAC register)	No commonly misidentified lines were used

Palaeontology and Archaeology

Specimen provenance	
Specimen deposition	
Dating methods	

☐ Tick this box to confirm that the raw and calibrated dates are available in the paper or in Supplementary Information.

Ethics oversight	

Note that full information on the approval of the study protocol must also be provided in the manuscript.

Animals and other organisms

Policy information about studies involving animals; ARRIVE guidelines recommended for reporting animal research

Laboratory animals	
Wild animals	
Field-collected samples	
Ethics oversight	

Note that full information on the approval of the study protocol must also be provided in the manuscript.

Human research participants

Policy information about studies involving human research participants

Population characteristics	
Recruitment	
Ethics oversight	

Note that full information on the approval of the study protocol must also be provided in the manuscript.

Clinical data

Policy information about clinical studies

All manuscripts should comply with the ICMJE guidelines for publication of clinical research and a completed CONSORT checklist must be included with all submissions.

Clinical trial registration	
Study protocol	
Data collection	
Outcomes	

Dual use research of concern

Policy information about dual use research of concern

Hazards

Could the accidental, deliberate or reckless misuse of agents or technologies generated in the work, or the application of information presented in the manuscript, pose a threat to:

No	Yes	
☒	☐	Public health
☒	☐	National security
☒	☐	Crops and/or livestock
☒	☐	Ecosystems
☒	☐	Any other significant area

Experiments of concern

Does the work involve any of these experiments of concern:

No	Yes	
☒	☐	Demonstrate how to render a vaccine ineffective
☒	☐	Confer resistance to therapeutically useful antibiotics or antiviral agents
☒	☐	Enhance the virulence of a pathogen or render a nonpathogen virulent
☒	☐	Increase transmissibility of a pathogen
☒	☐	Alter the host range of a pathogen
☒	☐	Enable evasion of diagnostic/detection modalities
☒	☐	Enable the weaponization of a biological agent or toxin
☒	☐	Any other potentially harmful combination of experiments and agents

ChIP-seq

Data deposition

☐ Confirm that both raw and final processed data have been deposited in a public database such as GEO.

☐ Confirm that you have deposited or provided access to graph files (e.g. BED files) for the called peaks.

Data access links<br>*May remain private before publication.*	
Files in database submission	
Genome browser session<br>(e.g. UCSC)	

Methodology

Replicates	
Sequencing depth	
Antibodies	
Peak calling parameters	
Data quality	
Software	

Flow Cytometry

Plots

Confirm that:

☒ The axis labels state the marker and fluorochrome used (e.g. CD4-FITC).

☒ The axis scales are clearly visible. Include numbers along axes only for bottom left plot of group (a 'group' is an analysis of identical markers).

☒ All plots are contour plots with outliers or pseudocolor plots.

☒ A numerical value for number of cells or percentage (with statistics) is provided.

Methodology

Sample preparation	Cells were trypsinized and washed 2 times with PBS 1X. They were then fixed with cold 70% ethanol and stored at -20°C until staining with propidium iodide (PI). Cells were centrifuged 5 min at 1200 rpm and washed 2 times with PBS 1X. Then, cells were resuspended in 300 µL of PI working solution (50 µg/ml of propidium iodide and 50 µg/ml of Rnase A in PBS) then incubated for 30 min in darkness and analyzed by flow cytometry using a FACS Calibur II
Instrument	FACS Calibur II
Software	Modfit Software Version 3.2.1, CellQuest Version 3.2
Cell population abundance	A total of 10,000 live, single cells were recorded for each sample

Gating strategy	Total cells were first gated according to their intrinsic size (FSC) and granularity (SSC) properties on a log scale. The gate excluded cellular debris from living cells. Living cells were then gated based on the area (FL2-A) and the width (FL2-W) of the signal for DNA staining to exclude doublets.

☒ Tick this box to confirm that a figure exemplifying the gating strategy is provided in the Supplementary Information.

Magnetic resonance imaging

Experimental design

Design type	
Design specifications	
Behavioral performance measures	

Acquisition

Imaging type(s)	
Field strength	
Sequence & imaging parameters	
Area of acquisition	

Diffusion MRI ☐ Used ☐ Not used

Preprocessing

Preprocessing software	
Normalization	
Normalization template	
Noise and artifact removal	
Volume censoring	

Statistical modeling & inference

Model type and settings	
Effect(s) tested	

Specify type of analysis: ☐ Whole brain ☐ ROI-based ☐ Both

Statistic type for inference (See Eklund et al. 2016)	
Correction	

Models & analysis

n/a	Involved in the study
☐	☐ Functional and/or effective connectivity
☐	☐ Graph analysis
☐	☐ Multivariate modeling or predictive analysis

Functional and/or effective connectivity	
Graph analysis	
Multivariate modeling and predictive analysis	

This checklist template is licensed under a Creative Commons Attribution 4.0 International License, which permits use, sharing, adaptation, distribution and reproduction in any medium or format, as long as you give appropriate credit to the original author(s) and the source, provide a link to the Creative Commons license, and indicate if changes were made. The images or other third party material in this article are included in the article's Creative Commons license, unless indicated otherwise in a credit line to the material. If material is not included in the article's Creative Commons license and your intended use is not permitted by statutory regulation or exceeds the permitted use, you will need to obtain permission directly from the copyright holder. To view a copy of this license, visit http://creativecommons.org/licenses/by/4.0/
